# Access, Utilization, and Quality of Behavioral Health Integration in Medicaid Managed Care

**DOI:** 10.1001/jamahealthforum.2023.4593

**Published:** 2023-12-28

**Authors:** K. John McConnell, Sara Edelstein, Jennifer Hall, Anna Levy, Maria Danna, Deborah J. Cohen, Jürgen Unützer, Jane M. Zhu, Stephan Lindner

**Affiliations:** 1 Center for Health Systems Effectiveness, Oregon Health & Science University, Portland; 2 Department of Family Medicine, Oregon Health & Science University, Portland; 3 Department of Psychiatry & Behavioral Sciences, University of Washington, Seattle; 4 Division of General Internal Medicine, Oregon Health & Science University, Portland

## Abstract

**Importance:**

Many states have moved from models that carve out to those that carve in or integrate behavioral health in their Medicaid managed care organizations (MCOs), but little evidence exists about the effect of this change.

**Objective:**

To assess the association of the transition to integrated managed care (IMC) in Washington Medicaid with health services use, quality, health-related outcomes, and measures associated with social determinants of health.

**Design, Setting, and Participants:**

This cohort study used difference-in-differences analyses of Washington State’s 2014 to 2019 staggered rollout of IMC on claims-based measures for enrollees in Washington’s Medicaid MCO. It was supplemented with interviews of 24 behavioral health agency leaders, managed care administrators, and individuals who were participating in the IMC transition. The data were analyzed between February 1, 2023, and September 30, 2023.

**Main Outcomes and Measures:**

Claims-based measures of utilization (including specialty mental health visits and primary care visits); health-related outcomes (including self-harm events); rates of arrests, employment, and homelessness; and additional quality measures.

**Results:**

This cohort study included 1 454 185 individuals ages 13 to 64 years (743 668 female [51.1%]; 14 306 American Indian and Alaska Native [1.0%], 132 804 Asian American and Pacific Islander [9.1%], 112 442 Black [7.7%], 258 389 Hispanic [17.8%], and 810 304 White [55.7%] individuals). Financial integration was not associated with changes in claims-based measures of utilization and quality. Most claims-based measures of outcomes were also unchanged, although enrollees with mild or moderate mental illness experienced a slight decrease in cardiac events (−0.8%; 95% CI, −1.4 to −0.2), while enrollees with serious mental illness experienced small decreases in employment (−1.2%; 95% CI −1.9 to −0.5) and small increases in arrests (0.5%; 95% CI, 0.1 to 1.0). Interviews with key informants suggested that financial integration was perceived as an administrative change and did not have substantial implications for how practices delivered care; behavioral health agencies lacked guidance on how to integrate care in behavioral health settings and struggled with new contracts and regulatory policies that may have inhibited the ability to provide integrated care.

**Conclusions and Relevance:**

The results of this cohort study suggest that financial integration at the MCO level was not associated with significant changes in most measures of utilization, quality, outcomes, and social determinants of health. Additional support, including monitoring, training, and funding, may be necessary to drive delivery system changes to improve access, quality, and outcomes.

## Key Points

**Question:**

How is integrating physical and mental health within Medicaid managed care associated with measures of utilization, quality, and outcomes, and rates of arrests, employment, and homelessness?

**Findings:**

In this cohort study of 1 454 185 Medicaid enrollees in Washington State between 2014 and 2019, a staggered rollout of financial integration was not associated with significant changes in claims-based measures of utilization or health-related outcomes.

**Meaning:**

The study results suggest that financial integration at the Medicaid managed care plan level may be insufficient to drive large delivery system changes in the short run.

## Teaser

This cohort study examines the association of the transition to integrated managed care in Washington State Medicaid with health services use, quality, health-related outcomes, and measures associated with social determinants of health.

## Introduction

In response to an ongoing mental health crisis, Medicaid programs have emphasized the benefits of integrating physical and behavioral health care, a strategy prioritized by recent initiatives from the Biden Administration and the US Department of Health and Human Services.^1,2^ To facilitate integration, states have moved their Medicaid managed care plans from a traditional carve-out model to a carved-in or integrated model. Within a carve-out model, the primary managed care organization (MCO) is responsible for physical health, while a separate behavioral health organization manages behavioral health services. Historically, carve outs were seen as a favorable way to control costs and ensure access to behavioral health services.^3^ However, success with clinical integration models^4,5,6,7,8,9,10,11,12^ led many states to reconsider the potential for financially integrated managed care to create a more seamless, whole-person approach. Financial integration has the potential to improve care by eliminating separate payers and networks and other barriers to care, such as separate credentialing and empaneling of clinicians. Integration may also create opportunities for MCOs to leverage data to support case management and may encourage MCOs to pay clinicians for care coordination and care collocation.^13^ To the extent that integration supports whole-person care, it may facilitate broader assessments of physical, mental, and social needs, potentially addressing issues associated with contacts with the justice system or housing instability.

Movement from the traditional carve-out model to an integrated carve-in model represents substantial change in how mental health care is financed and delivered for millions of Medicaid enrollees, a population with a disproportionately high prevalence of serious mental illness (SMI).^14^ In 2019, only 6 states maintained managed care carve-out contracts^15,16^ compared with 20 in 2004.^17^ Despite these shifts, there is a lack of evidence on the effects of financial integration within Medicaid managed care organizations, with most studies based on systems and data that preceded the Affordable Care Act.^15^

This study used a natural experiment to assess the outcomes of financial integration of behavioral and physical health in Medicaid MCOs. We analyzed changes in Washington State, which launched its Integrated Managed Care (IMC) initiative in 2016. This initiative shifted from a system with separate financing for behavioral and physical health to an integrated model, with the MCO responsible for payment for behavioral and physical health. The state of Washington transitioned to financial integration in a staggered fashion, with county groups moving from carve-out to carve-in models at 5 different times, with all regions transitioning by 2020.

We assessed the association between financial integration at the managed care level and a set of claims-based measures of health services use, quality, and health-related outcomes. We also tested the association of integrated care with measures associated with social determinants of health, including rates of homelessness, arrests, and employment, reflecting the increased interest by states in these measures and the hypothesis that if integration improves overall health, it could translate to improvements in these broader measures of well-being. We conducted separate analyses for (1) enrollees with SMI, (2) enrollees with mild or moderate mental illness (MMI), and (3) enrollees classified as having no mental illness, anticipating that the IMC effort would have heterogeneous associations with these populations.^18,19^ We supplemented the quantitative analyses with qualitative interviews, providing context of the on-the-ground experience of the IMC implementation.

## Methods

### Study Design and Setting

The study protocol was approved by the Washington State institutional review board. We followed the Strengthening the Reporting of Observational Studies in Epidemiology (STROBE) reporting guidelines.^20^ This study used Medicaid claims data and qualitative interviews with key stakeholders in Washington. Claims data were obtained from the Washington Health Care Authority. The enrollee study population included individuals ages 13 to 64 years who were enrolled in Washington’s Medicaid MCOs between January 1, 2014, and December 31, 2019. We excluded enrollees who were dually eligible for Medicare and Medicaid; part of the Emergency Medicaid or medically needy spend-down program; moved from one county grouping to another; or were part of the fee-for-service population. Enrollee-calendar years with fewer than 3 months of enrollment were also excluded. Self-reported data on race and ethnicity were included as part of the administrative data collection process. Additional details are provided in eMethods 1 in Supplement 1.

We defined enrollees with SMI as those with any inpatient or psychiatric residential claim or at least 2 other claims on separate dates during 1 year with a primary diagnosis of schizophrenia (*International Classification of Diseases, Ninth Revision [ICD-9]*/*ICD-10* 295.x; F20, F25), bipolar I (*ICD-9/10* 296.0, 296.1,296.4 − 296.7; F30, F31.0-F31.78), or major depressive disorder (*ICD-9/10* 296.2x, 296.33, 296.34; F32.2, F32.3, F33.2, F33.3). Enrollees in MMI were defined analogously (any inpatient or residential claim or at least 2 other claims) for mental health diagnoses not included in the SMI definition. Enrollees with no mental illness comprised the remainder of the study population (eAppendix 1 in Supplement1 ).

### Outcome Variables

We analyzed 5 measures of utilization (outpatient mental health visits, primary care visits for mental health, primary care visits for physical health, emergency department [ED] visits for mental health conditions or self-harm incidents, and inpatient/residential stays for mental health conditions); 4 health outcome measures (readmissions after mental health hospitalizations, cardiac events resulting in inpatient or ED visits, inpatient stays associated with diabetes, and self-harm incidents); and 3 outcomes associated with social determinants of health (any arrests, any employment, and any homelessness). The unit of observation for these outcomes was the person-quarter.

We also included several Healthcare Effectiveness Data and Information Set (HEDIS)^21^ quality measures for individuals with SMI: comprehensive diabetes care (hemoglobin A_1c_ testing only); monitoring of persistent medications; diabetes screening for people with schizophrenia or bipolar disorder who were using antipsychotic medication; and antidepressant medication management (acute and continuous). The unit of observation for HEDIS measures was the person-year.

By 2019, the US Centers for Medicare & Medicaid Services identified 5 codes that clinicians could use to bill for collaborative care (CoCM): *Current Procedural Terminology* codes 99492, 99493, and 99494 and Healthcare Common Procedure Coding System codes G0511 and G0512. These codes offer a mechanism to be paid directly for time spent coordinating behavioral and physical health (rather than treating integration as something that happens seamlessly without a need for additional resources). We analyzed their claim frequency and the percentage of total outpatient mental claims they represented.

### Statistical Analysis

The analyses of utilization measures and outcomes were conducted at the member-quarter level. The analyses of HEDIS measures were conducted at the member-year level.

Washington counties transitioned to IMC in 5 groups. The first group transitioned in 2016. Because preliminary analyses indicated that its prepolicy trends were significantly different than other groups and because a separate article assessed changes that isolated the first group,^18^ we omitted these counties in our main analysis. We tested the implications of this group’s inclusion in sensitivity analyses. We defined groups 2, 3, and 4 as treated groups; group 5 did not transition to IMC until 2020 and served as an untreated comparison group.

Our primary analysis was a difference-in-differences (DD) model with staggered treatment timing, with group 2 transitioning in January 2018, group 3 in January 2019, and group 4 in July 2019. We used data from 2014 to 2019, allowing for at least 12 quarters of preintervention data for each group, with 8 quarters of postintervention data for group 2, 4 quarters of postintervention data for group 3, and 2 quarters of postintervention data in group 4. We included the following covariates: age, sex, and the Chronic Illness and Disability Payment System risk adjusters.^22^

To account for the staggered implementation of IMC, we used a DD method developed by Sun and Abraham^23^ that accommodates heterogeneous group effects and differential timing of the intervention across groups. To minimize any effects of differential pre-IMC trends, we detrended our outcome variables, using the coefficient from the initial parallel-trend test to first detrend outcomes for treated groups. In sensitivity analyses, we tested the implications of using outcomes that were not detrended. All statistical analyses were performed using R statistical software, version 3.4.1 (R Project for Statistical Computing). Statistical significance was defined as 2-sided *P* < .05. A detailed description is provided in eMethods 2 in Supplement 1.

### Qualitative Data Collection and Analysis

Interviews were conducted between October 2021 and April 2023 using a semistructured guide. Regional leaders helped identify individuals who were knowledgeable about IMC. We purposively selected 24 participants from those referrals to ensure that we interviewed people who represented all regions and MCOs, following Standards for Reporting Qualitative Research reporting guidelines.^24^ Participants were asked about their experiences in transitioning to the carve-in model and the association IMC had with the ability to deliver integrated services to patients. We used an inductive data coding approach, using data from early interviews to inform subsequent sampling, interview guide questions, and monitor for saturation. Following coding, the team identified emerging preliminary findings and implications from the analysis using immersion crystallization methods.^25^ Interview guides and additional methodological details are provided in eAppendix 2 in Supplement 1.

## Results

### Effects of IMC

The study population included 1 454 185 enrollees. Table 1 displays enrollee demographic characteristics of the treatment and mental illness groups, with treatment groups generally similar to the comparison group. Table 2 displays results from the DD analyses on utilization metrics across all 3 cohorts (SMI, MMI, and no mental illness). Across all 3 enrollee types, we found no significant changes in claims-based utilization measures. For example, before behavioral health integration, outpatient mental health visits among enrollees with SMI averaged 805.6 visits per 1000 member months; the transition to IMC was associated with a slight decrease in these visits (−33.9), but this was not statistically significant (95% CI, −106.1 to 38.2).

**Table 1.** Population Characteristics

Characteristic	Serious mental illness, %^a^		Mild/moderate mental illness, %^a^		No mental illness, %^a^	
	Treated groups	Comparison groups	Treated groups	Comparison groups	Treated groups	Comparison groups
No.	44 858	8141	166 975	30 711	517 376	81 736
Age, y						
13-18	12.9	12.4	24.9	23.3	20.7	17.0
19-34	35.3	36.2	37.2	36.3	37.9	36.4
35-54	37.4	39.2	28.5	30.6	28.3	30.9
55-64	14.3	12.2	9.5	9.8	13.1	15.7
Female	57.4	59.1	60.0	60.4	49.4	47.9
Male	42.6	40.9	40.0	39.6	50.6	52.1
Race and ethnicity						
American Indian and Alaska Native	1.2	0.9	1.3	1.1	0.9	0.9
Asian American and Pacific Islander	5	2.5	4.4	2.3	11.2	5.6
Black	10.5	3.1	7.9	2.5	9.3	3.1
Hispanic	13.5	7.8	17.8	9.1	21.4	10.8
White	63.5	81.6	62.3	80.6	48.7	72.9
Other/missing	6.3	4.1	6.4	4.4	8.5	6.6
Risk factors						
Substance use disorder	26.4	23.7	14.9	14.2	4.8	6.1
Cancer	1.9	1.7	1.5	1.5	1.3	1.4
Cardiovascular conditions	26.2	23.6	17.3	18.2	12.7	14.4
Diabetes	10	9.7	5.7	6.2	5.2	5.3
Gastroenterological conditions	20.7	18.9	14.3	14.6	7.9	8.3
Pulmonary conditions	21.5	21.2	15.6	16.5	7.8	9.1

^a^ Values taken in quarter 4 of 2017 or the latest quarter available in that year. Risk factors are Chronic Illness and Disability Payment System risk adjusters.

**Table 2.** Utilization Outcomes From Difference-in-Differences Analyses

Measure	SMI		Mild and moderate mental illness		No mental illness^a^	
	Baseline	Estimate (95% CI)	Baseline	Estimate (95% CI)	Baseline	Estimate (95% CI)
Outpatient mental health visits	805.6	−33.9 (−106.1 to 38.2)	310.7	−12.5 (−42.3 to 17.3)	0.7	−0.13 (−0.32 to 0.05)
Primary care visits: mental health	57.4	−3.1 (−11.9 to 5.8)	47.2	0.4 (−1.4 to 2.3)	1.7	−0.11 (−0.27 to 0.06)
Primary care visits: physical health	307.9	−1.1 (−13.7 to 11.5)	262.0	1.1 (−6.4 to 8.5)	152.9	−1.4 (−5.6 to 2.8)
ED visits for mental health conditions	17.7	0.1 (−2.4 to 2.7)	3.8	−0.2 (−0.6 to 0.2)	0.2	−0.0 (−0.1 to 0.1)
Hospitalizations for mental health conditions^b^	15.8	1.8 (−4.0 to 7.6)	0.9	0.47 (−0.02 to 0.96)	NA	NA

Abbreviations: ED, emergency department; NA, not applicable; SMI, severe mental illness.

^a^ The no mental illness population was defined as enrollees without at least 1 inpatient visit with a mental health diagnosis or at least 2 other visits with a mental health diagnosis. Thus, it is possible for enrollees classified as having no mental illness to still have an outpatient mental health visit, primary care mental health visit, or ED visit for a mental health condition.

^b^ Unit of observation is the person quarter. Measures are presented as rates per 1000 member months.

Table 3 displays results from the DD analyses on outcomes and social determinants of health–associated measures for enrollees with SMI and MMI and quality measures for enrollees with SMI. Among enrollees with SMI, IMC was associated with a 0.9 percentage point decrease in employment (95% CI, −1.5 to −0.2) from a baseline rate of 30.9% and a 0.5 percentage point increase in arrests (95% CI, 0.1-1.0). Among enrollees with MMI, IMC was associated with a small reduction in cardiac events (−0.9; 95% CI, −1.5 to −0.3). However, IMC was not associated with most measures, including any changes in health outcomes or quality measures for enrollees with SMI.

**Table 3.** Health, SDOH, and Quality Outcomes From Difference-in-Differences Analyses

Characteristic^a^	SMI		Mild and moderate mental illness	
	Baseline	Estimate (95% CI)	Baseline	Estimate (95% CI)
Health outcomes				
Readmission after mental health hospitalization	23.8	−3.1 (−10.3 to 4.0)	9.3	3.9 (−1.2 to 9.1)
Self-harm incidents	4.3	0.1 (−0.6 to 0.8)	1.0	−0.1 (−0.3 to 0.1)
Cardiac events (IP or ED)	9.1	0.2 (−1.6 to 2.0)	5.1	−0.9 (−1.5 to −0.3)^a^
Hospitalizations for diabetes	63.7	13.6 (−16.3 to 43.5)	38.1	−18.0 (−37.8 to 1.8)
SDOH related				
Employed	30.9	−0.9 (−1.5 to −0.2)^a^	42.1	−0.6 (−1.3 to 0.1)
Arrested	4.3	0.5 (0.01 to 1.0)^a^	3.1	0.2 (−0.1 to 0.5)
Homeless	15.2	0.2 (−0.3 to 0.7)	9.1	−0.1 (−0.4 to 0.3)
Quality measures				
Comprehensive diabetes care: HbA_1c_	83.2	−0.1 (−3.0 to 2.9)	NA	NA
Monitoring of persistent medications	86.9	−0.9 (−4.4 to 2.7)	NA	NA
Diabetes screening for people using antipsychotics	78.0	1.2 (−1.4 to 3.8)	NA	NA
Antidepressant medication management: acute	47.3	2.6 (−3.2 to 8.4)	NA	NA
Antidepressant medication management: continuation	31.5	2.0 (−2.4 to 6.5)	NA	NA

Abbreviations: ED, emergency department; HbA_1c_, hemoglobin A_1c_; IP, inpatient; NA, not applicable; SDOH, social determinants of health; SMI, severe mental illness.

^a^ Unit of observation for health outcomes and SDOH measures is the person quarter. Measures are presented as rates per 1000 member months, with 1 exception: hospitalizations for diabetes are shown as rates per 100 000 member months. The unit of observation for quality measures is the person year.

We completed a total of 24 interviews with key stakeholders, including 10 interviews with community leaders who participated in the IMC transition, 8 interviews with behavioral health agencies (BHAs), and 6 interviews with MCO administrators. Interviewees described the transition to IMC as an administrative change but not a care delivery change. Participants reported that the transition to IMC was not associated with how primary care clinics or BHAs delivered care:

To my knowledge, there haven’t been any changes at the level of clinical integration. I’ve truly envisioned this stage of integrated managed care as financial integration, and that’s it. […] I haven’t seen any evidence that there have been any improvements with clinical integration (group 5, participant 2, community leader).

Participants noted that primary care clinics already had contracts in place with MCOs, with the transition to IMC having few implications for these clinics. In contrast, IMC required BHAs to establish new contracts and relationships with MCOs, adopt new electronic health records to meet MCO documentation and billing requirements, and change their reporting and billing procedures. Participants reported that MCO contracts either were not revised to reimburse BHAs for integrated services or took time to change, and modified payment models were needed to accommodate the SMI population’s primary care needs. These new MCO contracts often required revisions to receive reimbursement for primary care in behavioral health settings.

My experience has been that it takes maybe 6 months or a year to get to an [MCO] decision on updating an agreement.[..] and we’ll have to do it 5 different times (group 1, participant 13, BHA).

The BHAs also indicated that they lacked support, guidance, and technical assistance in identifying appropriate integrated care models, particularly in adopting a model aligned with their organization’s size and scope. Participants noted that regulatory and licensing restrictions limited opportunities for integration. For example, BHA licensure required a full intake before service provision, limiting the ability to offer brief therapy interventions that might have facilitated the integration of physical and behavioral health care. Interviewees suggested that revisions to regulatory policies would be needed to implement emerging models of integrated care.

I think [the state] needs to think about how they can change some of their [administrative code], whatever’s in place that might be a hindrance to integration. Some of that might be requiring lobbying up to the federal government around maybe some of the rules (group 1, participant 12, community leader).

### CoCM Codes

Coordinated care model codes were rarely used in claims for our study population. They first appeared in 2018 and were associated with only 1694 of 3 216 086 outpatient specialty or primary care mental health claims (0.05%) from 2018 to 2019, suggesting relatively little uptake.

### Sensitivity Analyses

We conducted 3 sensitivity analyses: (1) the DD approach described previously, with group 1 included as a fourth treatment group; (2) DD without detrending rates, with group 1 excluded; and (3) DD without detrending rates, with group 1 included. Results were qualitatively similar to the primary analyses. Additional details are provided in eMethods 3 in Supplement 1.

## Discussion

In this cohort study, we examined the outcomes of staggered financial integration of behavioral and physical health care in Washington State’s Medicaid managed care agencies. We tested a comprehensive set of measures associated with utilization, outcomes, quality, and social determinants of health. The transition to IMC was not associated with significant changes in most outcomes nor accompanied by substantial increases in the use of CoCM codes. Informant discussions indicated that IMC primarily affected the structure and financing of managed care, not the delivery system. In particular, IMC appeared to have little association with primary care clinics, which maintained their ongoing contracts with MCOs, but may have been disruptive for BHAs, some of whom reported struggles in setting up new contracts and electronic health records.

The study results aligned with a 2020 qualitative review commissioned by the National Council for Behavioral Health^26^ that found that carve ins were not uniformly associated with integration at the delivery system level. In many states, financial integration lacked accompanying efforts to address systemic barriers, like inadequate investment in health information technology, lack of financial reserves in BHAs, and insufficient administrative infrastructures to handle increased demands from multiple managed care plans.

We also found limited adoption of CoCM codes, which was consistent with other studies and in line with findings that suggested a substantial time lag between the availability of new billing codes for CoCM and the widespread use of such codes in practice.^27,28,29,30,31,32,33,34,35,36^ The low uptake in Washington State is noteworthy, given the presence of the University of Washington’s Advancing Integrated Mental Health Solutions Center, which works to advance the implementation of CoCM. Even with local facilitation available, changes at the administrative or billing levels may be insufficient to drive short-term improvements in the provision of mental health services.

Although we did not find evidence of positive changes, financial integration was not associated with adverse effects. This finding contradicts earlier literature that found that carve-out models were more likely to be associated with improved access.^3^ Moreover, financial integration may have been associated with reduced complexity for enrollees without sacrificing access, and expected positive changes may take longer to be realized.

While financial integration may not be sufficient to improve mental health outcomes, it may be a necessary first step, allowing states to layer additional training, incentives, and supports onto a single accountable plan. Washington is pursuing this approach with an integrated care assessment initiative,^37^ collecting data to support policy and funding decisions. These efforts may provide mechanisms to address some of the barriers to integration, including the lack of technical assistance and outdated regulations, that were identified in our qualitative interviews.

States aiming for clinical integration may need to combine financial integration with investments in workforce recruitment and training and strengthen contracting and data analytics expertise for performance monitoring and oversight.^38^ Arizona’s move to a carve-in model was accompanied by a targeted investments program, representing a major investment designed to foster collaboration between clinicians and develop information-sharing tools, data analysis standards, and protocols to enable managing and coordinating patient care across multiple clinicians.^39^ The program incentivized clinicians to achieve milestones, including integrated care plans and behavioral health screening in primary care, and in a later phase, performance on selected HEDIS metrics, accompanied by data from an integrated practice assessment tool.^40^ The state has reported substantial increases in the share of practices that have advanced clinical integration.^39^ Future research could assess whether these efforts are associated with improved care coordination and health-related outcomes.

### Limitations

This study had several limitations. Our study may be limited in the relatively short post-IMC periods, particularly given the delays in contracting described by BHAs. We observed at most 8 postintervention quarters, and only 4 quarters for group 3, the largest of the county groups. A separate study of access measures in IMC focused exclusively on group 1, allowing for 15 quarters (almost 4 years) of postintervention observations.^18^ That study found a slight increase in access to primary care (an increase of 2 percentage points from a baseline of 61%) but no changes in access to outpatient mental health services or the use of hospital or ED services for mental health. Our claims data may not be a full representation of all behavioral health encounters during the study period, and it is possible that clinical measures changed even if claims-based measures did not. Despite the large sample size, our confidence intervals were large, and we may not have precisely detected some changes that occurred with integration. Our interviews were conducted from 2021 to 2023, meaning that respondents had to retrospectively describe the transition; this may have introduced recall bias.

## Conclusions

In this cohort study, the transition to the integration of physical and behavioral health in Washington Medicaid managed care was not associated with significant changes in claims-based measures of utilization or quality. Financial integration may be a necessary but insufficient first step for clinical integration. Additional contracting requirements, incentives, data, and training may be needed to drive delivery system change and achieve improvements in access and patient outcomes.

## Notes

Supplement 1. eAppendix 1. Counties by Group eMethods 1. Cohort Selection; Population/Measure Definitions; Regression eAppendix 2. Washington State Integrated Managed Care Interview Guide eMethods 2. Qualitative Methods eAppendix 3. Event Study Graphs eMethods 3. Sensitivity Analyses Results

Supplement 2. Data sharing statement
