# Supplementary material for: Access, Utilization, and Quality of Behavioral Health Integration in Medicaid Managed Care
Source: JAMA Health Forum. 2023 Dec 28;4(12):e234593. doi: 10.1001/jamahealthforum.2023.4593 (PMC10755612; doi:10.1001/jamahealthforum.2023.4593)
Supplement: Supplement 1. — eAppendix 1. Counties by Group eMethods 1. Cohort Selection; Population/Measure Definitions; Regression eAppendix 2. Washington State Integrated Managed Care Interview Guide eMethods 2. Qualitative Methods eAppendix 3. Event Study Graphs eMethods 3. Sensitivity Analyses Results [file jamahealthforum-e234593-s001.pdf]

Supplemental Online Content

McConnell KJ, Edelstein S, Hall J, et al. Access, utilization, and quality of behavioral health integration in Medicaid managed care. *JAMA Health Forum*. 2023;4(12):e234593.
doi:10.1001/jamahealthforum.2023.4593

eAppendix 1. Counties by Group

eMethods 1. Cohort Selection; Population/Measure Definitions; Regression

eAppendix 2. Washington State Integrated Managed Care Interview Guide

eMethods 2. Qualitative Methods

eAppendix 3. Event Study Graphs

eMethods 3. Sensitivity Analyses Results

This supplemental material has been provided by the authors to give readers additional information about their work.

© 2023 McConnell KJ et al. JAMA Health Forum.

eAPPENDIX 1 Counties by IMC Transition Group

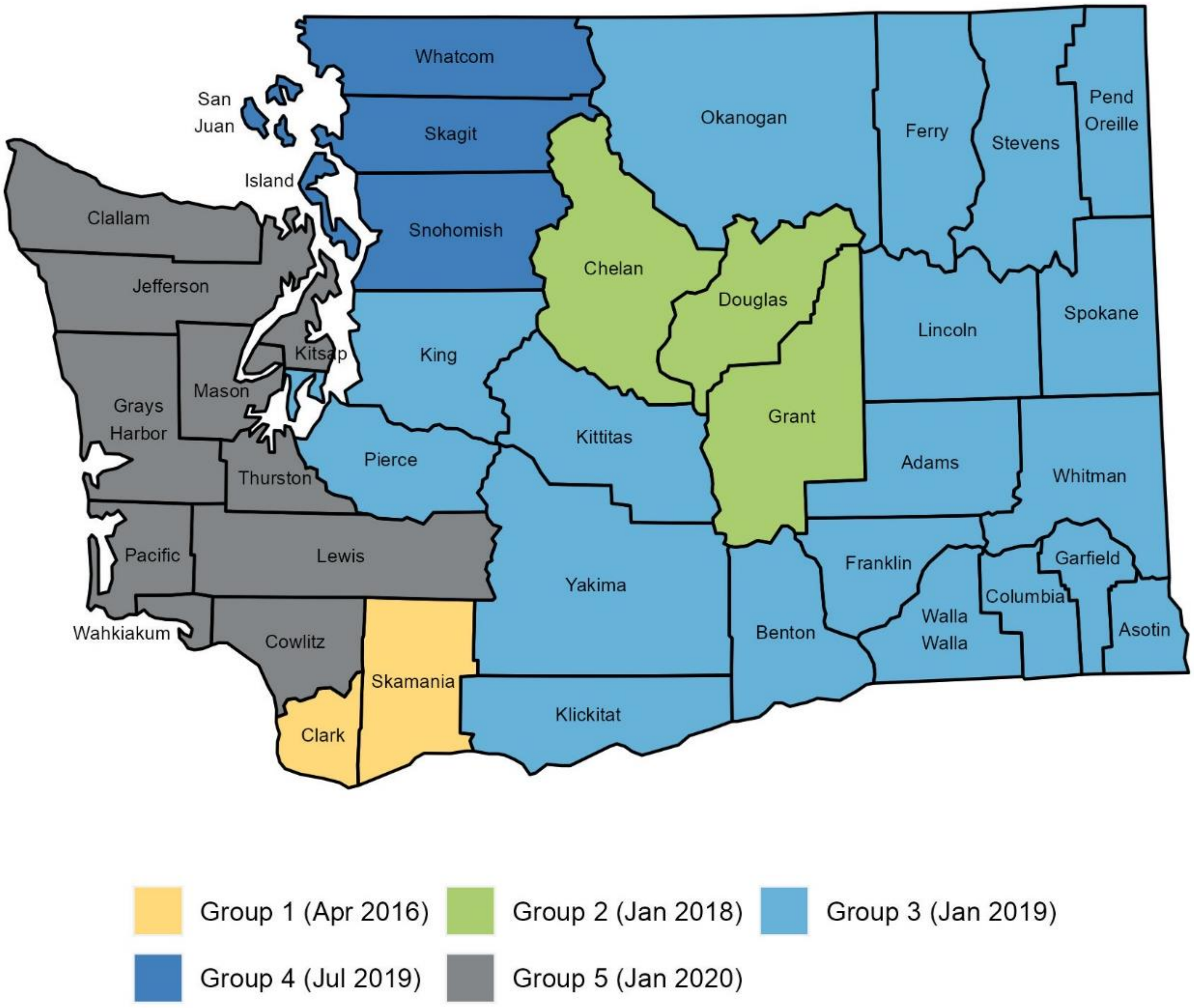

© 2023 McConnell KJ et al. JAMA Health Forum.

eMETHODS 1

Cohort Selection and Population/Measure Definitions

Identifying sample population

We obtained data on Medicaid beneficiaries enrolled in 2014 through the first half of 2021. Members were dropped if they ever had an inconsistent age recorded within or across quarters. Then all quarters in which a member was less than 13 years old or more than 65 years old were dropped. Members were then dropped if they had any months in which they were a medically needy spend-down enrollee, a pregnant non-citizen enrollee, or dually eligible for Medicare.

We further dropped members with more than three months of enrollment in fee-for-service rather than managed care, or more than three months with missing county date, as county is used to determine when the timing of transition to IMC. We allowed for moving county within IMC group, but dropped members who ever switched IMC group. We then dropped member-calendar years in which the member was not enrolled for at least three months. We dropped quarters beyond 2019. In the final step, we dropped county group one members, though they were included in the sensitivity analyses detailed in this appendix.

Sample Selection Step	Members	Member quarters	Members dropped (%)	Member quarters dropped (%)
Start	3,602,675	57,416,738		
Drop if ever inconsistent age	3,599,084	57,356,012	0.1	0.1
Drop age < 13 or age > 65	2,548,576	35,381,355	29.2	38.3
Drop if ever medically needy	2,524,222	34,938,110	1.0	1.3
Drop if ever pregnant non-citizen	2,485,440	34,567,936	1.5	1.1
Drop if ever dually eligible	2,339,876	31,805,834	5.9	8.0
Drop if 4+ months not in managed care	1,990,857	26,886,269	14.9	15.5
Drop if 4+ months with invalid county code	1,990,229	26,880,365	0.0	0.0
Drop if person ever switches IMC group	1,842,105	24,100,862	7.4	10.3
Drop year if enrolled < 3 months in year	1,785,554	23,572,230	3.1	2.2
Limit to 2014-2019	1,563,060	18,273,898	12.5	22.5
Limit to county groups 2-5	1,454,185	17,065,660	7.0	6.6

Identifying Mental Illness

Individuals were included in the Serious Mental Illness (SMI) subpopulation if, during any 12-month period within the study period, they had one inpatient visit or two non-inpatient visits with a primary diagnosis of SMI. SMI diagnoses are as follows: Schizophrenia (ICD9 295, ICD10 F20, F25); Bipolar I (ICD9 296.0, 296.1,296.4−296.7, ICD10 F30, F31.0-F31.78); and Major Depressive Disorder (ICD 9 296.2, 296.33, 296.34, ICD 10 F32.2, F32.3, F33.2, F33.3).

Individuals were included in the Mild/Moderate Mental Illness (MMMI) subpopulation if, during any 12-month period within the study period, they had one inpatient visit or two non-inpatient visits with a primary diagnosis indicating a mental health condition or self-harm incident and did not meet the criteria for SMI.

© 2023 McConnell KJ et al. JAMA Health Forum.

Mental Health Condition: ICD9 295-298, 300-302, 306-309, 311-314 and subcodes, excluding 302.0, 307.0, 307.20-307.23, 307.3, 307.6, 307.7, 307.9, 309.21, 313.23, 313.89, 313.9 / ICD10 F20-69, F90-99 and subcodes, excluding F48.2, F55, F64.2, F93, F94, F95, F98. To arrive at these lists, first we chose broad ranges of ICD9/ICD10 codes for mental disorders, excluding mental disorders due to physiological conditions, substance use, intellectual disabilities, and developmental disorders. We then merged Clinical Classifications Software (CCS) categories onto these codes and dropped codes that had no CCS category (302.0) or fell in one of the following CCS categories: Delirium dementia and amnestic and other cognitive disorders, Developmental disorders, Disorders usually diagnosed in infancy, childhood, or adolescence, or Substance-related disorders. While Attention-deficit Hyperactivity Disorder (ADHD) and autism are both considered neurodevelopmental disorders, ADHD is included in our list while autism is not, as the former falls in the CCS category Attention-deficit, conduct, and disruptive behavior disorders and the latter falls in developmental disorders.

Self-Harm Incident: ICD9 E950–E958 / ICD10 codes that fall in the Clinical Classifications Software Refined categories MBD012 (Suicidal ideation/attempt/intentional self-harm) or EXT021 (External cause codes: intent of injury, self-harm), except for R45851 (Suicidal Ideation).

Outcome Definitions

Outcomes are defined as follows. All outcomes are converted to a per 1,000 member months estimate unless otherwise noted. Outpatient Specialty Mental Health Visits: Visits per quarter with a mental illness or self harm diagnosis, a CPT that could be used in a specialty mental health setting, an outpatient place of service, and either a provider or organization taxonomy code or a place of service indicating a mental health provider.

- Diagnosis: an ICD code indicating a mental health condition in the primary diagnosis field or a self-harm incident in the first four diagnosis codes or four injury codes (self harm codes are less likely to appear in the primary field).
- CPTs: 90791, 90792, 90820, 90832, 90833, 90834, 90835, 90836, 90837, 90838, 90839, 90840, 90845, 90846, 90847, 90849, 90853, 90863, 90875, 90876, 90880, 90882, 90887, 90899, 96101, 96102, 96103, 96116, 96118, 96119, 96120, 96127, 96130, 96131, 96132, 96133, 96136, 96137, 96150, 96151, 96152, 96153, 96154, 96155, 99201, 99202, 99203, 99204, 99205, 99211, 99212, 99213, 99214, 99215, 99241, 99242, 99243, 99244, 99245, 99381, 99382, 99383, 99384, 99385, 99386, 99387, 99391, 99392, 99393, 99394, 99395, 99396, 99397, 99401, 99402, 99403, 99404, 99411, 99412, 99420, 99429, 99441, 99442, 99443, 99484, 99492, 99493, 99494, G0074, G0502, G0503, G0504, G0507, H0002, H0031, H0032, H0034, H0036, H0038, H0046, H2010, H2011, H2015, H2016, H2019, H2020, H2021, H2022, H2023, H2024, H2025, H2026, H2027, H2030, H2031, H2032, H2033, J1631, J2794, S9482, S9484, S9485, T1015, T1016, T1017, T1023, T2022, T2023
- Place of Service: 2-3, 5-8, 11-15, 17, 19-20, 22, 49, 50, 53, 62, 71, 72, 99. (99 was included as these claims often had an NPI and CPT indicating an outpatient service). 53 (community mental health center) was used to identify mental health specialists in cases where taxonomy data was missing or ambiguous.
- Taxonomy:
  - Individuals: 101200000X, 101Y00000X, 101YA0400X, 101YM0800X, 101YP1600X, 101YP2500X, 101YS0200X, 102L00000X, 102X00000X, 103G00000X, 103GC0700X, 103T00000X, 103TA0400X, 103TA0700X, 103TB0200X, 103TC0700X, 103TC1900X, 103TC2200X, 103TE1000X, 103TE1100X, 103TF0000X, 103TF0200X, 103TH0004X, 103TH0100X, 103TM1700X, 103TM1800X, 103TP0016X, 103TP0814X, 103TP2700X, 103TP2701X, 103TR0400X, 103TS0200X, 103TW0100X, 104100000X, 1041C0700X, 1041S0200X, 106H00000X, 163WP0807X, 163WP0808X, 163WP0809X, 2084A0401X, 2084B0002X, 2084B0040X, 2084F0202X, 2084N0600X, 2084P0015X, 2084P0800X, 2084P0802X, 2084P0804X, 2084P0805X, 363LP0808X, 364SP0807X, 364SP0808X, 364SP0809X, 364SP0810X, 364SP0811X, 364SP0812X, 364SP0813X
  - Organizations: 251S00000X, 261QM0801X, 261QM0850X, 261QM0855X, 261QR0405X, 273R00000X, 283Q00000X, 320800000X, 323P00000X

© 2023 McConnell KJ et al. JAMA Health Forum.

Primary Care Mental Health Visits: Visits per quarter with a mental illness or self harm diagnosis, a CPT that could be used in a primary care setting, an outpatient place of service, and either a provider or organization taxonomy code indicating a primary care provider.

- Diagnosis: an ICD code indicating a mental health condition in the primary diagnosis field or a self-harm incident in the first four diagnosis codes or four injury codes.
- CPTs: 99201-99205, 99211-99215, 99241-99245, 99381-99387, 99391-99397, 99401-99404, 99411, 99412, 99420-99429
- Place of Service: 2-3, 5-8, 11-15, 17, 19-20, 22, 49, 50, 53, 62, 71, 72, 99.
- Taxonomy:
    - Individuals: 204C00000X, 204D00000X, 207Q00000X, 207QA0000X, 207QA0505X, 207QB0002X, 207QG0300X, 207R00000X, 207RA0000X, 207RB0002X, 207RG0300X, 208000000X, 2080A0000X, 2080B0002X, 2083B0002X, 2083P0500X, 2083P0901X, 208D00000X, 363A00000X, 363AM0700X, 363L00000X, 363LA2200X, 363LC1500X, 363LF0000X, 363LG0600X, 363LP0200X, 363LP2300X, 363LW0102X
    - Organizations: 261QF0400X, 261QR1300X, 261QP2300X

Hospitalizations for Mental Health Conditions: Visits per quarter to an inpatient hospital, psychiatric residential facility, or partial hospitalization program (as indicated by the claim's place of service or claim type) with a mental health condition in the primary diagnosis field or a self-harm diagnosis in the first four diagnosis codes or four injury codes.

Emergency Department Visits for Mental Health Conditions: Visits per quarter to an emergency department not followed by an inpatient hospitalization, with a mental health condition in the primary diagnosis field or a self-harm diagnosis in the first four diagnosis codes or four injury codes.

Readmission after Mental Health Hospitalization: Percentage of discharges from a hospitalization for a mental health condition (see above definition) followed by admission within 30 days to an inpatient hospital with any diagnosis, or to a psychiatric residential facility or partial hospitalization program with a primary diagnosis of a mental health condition. Limited to one discharge per member per month, allowing for up to three per quarter.

Self-Harm Incidents: Incidents of self-harm (see above definition) per quarter.

Cardiac Events: Inpatient or emergency department visits with a primary diagnosis of hypertension or a primary or secondary diagnosis of another cardiac condition. These diagnoses were flagged using the CCS categories 98-99 (hypertension) and 97, 100-101, 106-110, 112, 115, and 131 (other conditions).

Hospitalizations for Diabetes: Hospitalizations per quarter related to diabetes, based on Agency for Healthcare Research and Quality (AHRQ) Prevention Quality Indicators (PQI). Diabetes Short-term Complications Admissions (PQI-01), Diabetes Long-term Complications Admissions (PQI-03), Uncontrolled Diabetes Admissions (PQI-14), and Lower-Extremity Amputation among Patients with Diabetes (PQI-16) are included in the sum of hospitalizations. Reported as hospitalizations per 100,000 member months. Only includes enrollees ages 18 and older.

Comprehensive Diabetes Care (CDC) – Hemoglobin A1c (HbA1c) testing – HEDIS measure: Received any HbA1c testing in the past 12 months. Includes enrollees ages 18 and older.

Monitoring of Persistent Medications (MPM) – HEDIS measure: Received at least 180 treatment days of medication and at least one medication monitoring visit in the past 12 months. The included medications are ACE inhibitors or ARBs, Digoxin, and diuretics. Includes enrollees ages 18 and older.

© 2023 McConnell KJ et al. JAMA Health Forum.

Diabetes Screening for People With Schizophrenia or Bipolar Disorder Who Are Using Antipsychotic Medications (SSD) – HEDIS measure: Had a diabetes screening test in the past 12 months. Includes enrollees ages 18 and older with schizophrenia or bipolar disorder who were dispensed an antipsychotic medication.

Antidepressant Medication Management (AMM) – HEDIS measure: Remained on antidepressant medication for at least 12 weeks (acute measure)/at least 6 months (continuation measure). Includes enrollees ages 18 and older who were treated with antidepressant medication and had a diagnosis of major depression.

We used Washington's integrated client database (ICDB) to analyze the effect of integrated care on key public health outcomes. The ICDB includes the following outcomes.

Arrests are derived through data recorded in the Washington State Identification System, an arrest database maintained by the Washington State Patrol. The database is comprised of arrest charges for offenses resulting in fingerprint identification, providing a relatively complete record of felony and gross misdemeanor charges.

Homelessness is defined as an entry of "Homeless without Housing", "Emergency Shelter" or "Battered Spouse Shelter" recorded in the Economic Services Administration's Automated Client Eligibility System, which is used by caseworkers to record information about client self-reported living arrangements and shelter expenses when determining eligibility for cash, food, and medical assistance.

Employment is defined as having least one quarter in the measurement year with positive earnings recorded in the Employment Security Department's quarterly wage data

Regression Approach

We first determined effects for each group in each period pre- and post-intervention, by estimating the following model:

$$\boldsymbol{Y_{ijtq} = \beta_0 + GROUP_j + \sum_{q=2}^{q=24} \alpha_q + \sum_{t=-22}^{t=7} \gamma_t * \delta_t * GROUP_j + \theta X_{ijt} + \varepsilon_{ijt}}$$

where $\mathbf{Y_{ijt}}$ was the outcome of interest (e.g., OPMH visits) for individual ***i*** in county group **j**, in calendar quarter **q**, in relative quarter ***t*** (relative to IMC implementation, with the first quarter post-implementation as ***t*** = 0). **GROUPj** was an indicator for each of the three treated groups, with group five held out as a reference; and $\boldsymbol{\alpha_t}$ was an indicator for each of the 24 calendar quarters, with the first (Q1 2014) held out as a reference quarter. The coefficients $\boldsymbol{\gamma}$ represent the interaction between ***t*** and **GROUP.** Interaction effects existed only when a group was observed during a certain time; for example, group two had effects for ***t*** = -14 through ***t*** = 7, since its implementation occurred in the first quarter of 2018. Demographic covariates, including age, sex, race/ethnicity, and CDPS risk adjusters are captured in $\mathbf{X_{it}}$**;** ε_{ijt}, is a random error. For HEDIS outcomes, **q** was calendar year and ***t*** was relative year. Only groups two and three were included as treated groups.

Individual group-time effects in the post-intervention period were then aggregated, taking into account each group's size, to produce estimates for the effect of IMC overall, by group, and by quarter.

We conducted three sensitivity analyses: 1) methods described above with group one included; 2) DiD without detrending rates, group one excluded; 2) DiD without detrending rates, group one included. For #1 and #3 for the primary care visits outcome, the first quarter of 2014 was not included due to unusual trends for group one that were thought to be a data anomaly. Results of these tests can be found in Section D.

© 2023 McConnell KJ et al. JAMA Health Forum.

eAPPENDIX 2

Washington State Integrated Managed Care Interview Guide

Washington State Integrated Managed Care (IMC) Implementation
Interview Guide: IMC Transition Participants, Community Leaders

Introduction

Thank you for participating in this interview. We are speaking with you today because we value your perspective, and we'd like to hear about your experiences with IMC implementation in Washington. We are hoping to learn more about how integrated managed care has progressed and your perceptions on successes, challenges, and barriers that exist to IMC implementation.

- Introduce yourself and second interviewer if applicable.
- Did you have a chance to review the information sheet? Do you have questions?
- Describe how the recording interview transcripts are de-identified and handled:
    - Recordings will be professionally transcribed, and any information in the interview that could be used to identify you will be stripped from the transcripts. These transcripts will only be seen by and shared with the research team.

Start recording: Do I have your permission to record this interview?

---

1. **Please tell me about yourself.**
    a. What organization do you work for?
    b. What is your role and title?
    c. What was your role in [region's] transition to IMC?

2. **Please tell us about [insert region].**
    a. [Probe for size, geography, population]

3. **Thinking back to before IMC began, please describe how behavioral health care was organized and administered in [region]?**
   [Listen and probe for differences between mental health (previously managed by RSNs and SUD (previously managed by counties]
    a. What organizations were involved?
    b. What were the challenges with this structure?
    c. What were the benefits?

4. **Why did your region elect to become an [insert adopter]?**
    a. What influenced your decision?
    b. Who was part of that decision?
    c. Were there problems (with mental health access or delivery) in your region that IMC was intended to help?

I'd love hear, from your perspective, about your experience transitioning to IMC.

© 2023 McConnell KJ et al. JAMA Health Forum.

5. **Could you describe your region's experience transitioning to IMC for me?**
    a. What was involved in this transition?
    b. What did the work look like?
    c. What had to be done to transition?
    d. Who led the work?
        - What partners were involved?
        - What were their roles
            - What was the role of the BH-ASO (if applicable)
        - What kinds of decisions had to be made to move this work forward?
        - How were those decisions made?

6. **What barriers or challenges did you experience?**
    a. To what extent have these issues endured?

7. **What's gone well?**
    a. Why?

We understand this transition took a lot of work.

8. **Could you describe for me what aspects of IMC integration were accomplished before the IMC deadline in your region?**
    a. What work remained after the deadline?
    b. What work still remains?

Now I'd like to ask you about the impact of the integrated managed care transition.

9. **In general, what have been the impacts of integrated managed care?**
    a. What impact has IMC had on integration at the care delivery level?
    b. In what ways does IMC foster clinical integration? How so?
    c. What impact has IMC had on patients?c
    d. provider organizations to integrate care at the care delivery level?
    e. **Priority: What impact has IMC had on the social determinants of health**

10. **How has the transition to IMC impacted the provider organizations in your region?**
    a. Can you share some examples of this impact?
    b. Probe for: primary care practices, community mental health centers
    c. Probe [if they can't speak to it firsthand]: Can you share some examples of a time when the transition's impact on provider organizations came up in workgroups or meetings that you attended?

Now I'd like to discuss your experience with the IMC transition and how it has varied by county or by working with the different MCOs.

11. **How has your experience with the IMC transition varied by MCO, if at all?**
    a. In what ways has it varied?

12. **How has your experience with the IMC transition varied by county, if at all?**

© 2023 McConnell KJ et al. JAMA Health Forum.

a. In what ways has it varied?

13. **Before we close, what have we not asked about, but should know, about your experience related to integrated managed care?**

© 2023 McConnell KJ et al. JAMA Health Forum.

Washington State Integrated Managed Care (IMC) Implementation
Interview Guide: Behavioral Health Agency

Introduction
Thank you for participating in this interview. We are speaking with you today because we value your perspective, and we'd like to hear about your experiences with IMC implementation in Washington. We are hoping to learn more about how integrated managed care has progressed and your perceptions on successes, challenges, and barriers that exist to IMC implementation.

- Introduce yourself and second interviewer if applicable.
- Did you have a chance to review the information sheet? Do you have questions?
- Describe how the recording interview transcripts are de-identified and handled:
    - Recordings will be professionally transcribed, and any information in the interview that could be used to identify you will be stripped from the transcripts. These transcripts will only be seen by and shared with the research team.

Start recording: Do I have your permission to record this interview?

1. **Please tell me about yourself.**
    a. What organization do you work for?
    b. What is your role and title?
    c. What was your role in your organization's transition to IMC?

2. **Please tell me about your organization**
    a. Services you provide
    b. Ownership and size
    c. Patients you serve
    d. Local geography and community

3. **Could you describe your organization's experience transitioning to IMC for me?**
    a. When did you transition?
    b. What did your organization do to transition?
        i. What did the work look like?
    c. Who was involved?

4. **What barriers or challenges did you experience?**
    a. To what extent have these issues endured?
    b. Challenges with MCO contract negotiations?

5. **What went well?**
    a. Why?

6. **What impact has IMC had on your organization's ability to deliver integrated care?**
    a. Please describe the types of integrated services you provide.
    b. Can you help me understand exactly how IMC has influenced this integrated service?

c. What else influences your ability to deliver integrated care?

7. **What else is needed for your organization to provide integrated care?**
    a. What barriers exist?

8. **What impact has IMC had on crisis services?**
    a. How does care get coordinated between crisis services and other levels of care (outpatient services, hospitalization services) that are managed by MCOs?
    b. Can you give us an example of what this looks like?
    c. What issues/challenges/barriers are there with the current system?
    d. Could you tell us more about your organization's youth mobile crisis program?

9. **Could you tell me about the types of payment methodologies and contracts your organization has with MCOs?**
    a. How do these methodologies compare to what you had in place before IMC?
    b. To what extent do these contracts facilitate integrated care?
        i. How so?
    c. What types of contracts or contract modifications are needed to facilitate integrated care?
    d. **How has your experience with the IMC transition varied by MCO, if at all?**
        i. In what ways has it varied?
    e. Can you tell me about the payment methodologies and contracts your organization has with Beacon?
    f. What impact has IMC had on your organization's ability to pay for services?
        - Which services?
        - How specifically has IMC impacted this?
    - What impact has IMC had on your approach to staffing?
        - Has your practice experienced staffing changes?

10. **How does your organization finance the model of integrated care that you have?**
    i. What integrated services are billable services?
    ii. What services are not billable?
    b. What impact has IMC had on your approach to billing?
        i. What billing changes have you made?
        ii. To what extent do you use Coordinated Care model billing codes?
        iii. What influences your practices' use of CCM codes?

11. **What impact has IMC had on the services your organization delivers to patients?**
    a. What impact have the access to care standard changes had on the services you deliver?
    b. Could you describe any changes in the quality of care provided during patient visits post-IMC?
    c. How has IMC impacted communication between primary care and behavioral health providers when it comes to addressing the physical and mental health needs of their patients?
        i. Have you noticed any changes in screening for or addressing mental health concerns?

12. **What impact has IMC had on your patients?**
    a. For which patients?
    b. Have there been certain racial/ethnic groups or specific communities more impacted than others?
    c. Probes:
        i. Patients with SMI?

© 2023 McConnell KJ et al. JAMA Health Forum.

ii. Youth and adolescents?
iii. Other groups?

13. What impact has IMC had on your organization's ability to address the social determinants of health?
   a. How so?
   b. For which determinants?

14. Who else would you recommend we speak to in [County] to get a small community mental health practices' perspective and its impacts?
   - People who actually deliver health services

15. Before we close, what have we not asked about, but should know, about your experience related to integrated managed care?

© 2023 McConnell KJ et al. JAMA Health Forum.

Washington State Integrated Managed Care (IMC) Implementation
Interview Guide: MCOs

Introduction
Thank you for participating in this interview. We are speaking with you today because we value your perspective, and we'd like to hear about your experiences with IMC implementation in Washington. We are hoping to learn more about how integrated managed care has progressed and your perceptions on successes, challenges, and barriers that exist to IMC implementation.
As an MCO that's participated in the IMC transition in # regions, you have a really important perspective because you are uniquely positioned to speak to the similarities and differences across the regions. As we move through each of these questions, I'm hoping you can share about how your experience has varied by region.

- Introduce yourself and second interviewer if applicable.
- Did you have a chance to review the information sheet? Do you have questions?
- Describe how the recording interview transcripts are de-identified and handled:
    - Recordings will be professionally transcribed, and any information in the interview that could be used to identify you will be stripped from the transcripts. These transcripts will only be seen by and shared with the research team.
- Start recording: Do I have your permission to record this interview?

---

1. **Please tell me about yourself.**
    a. What is your role and title?
    b. What was your role in the transition?

2. **Please tell me a little about your MCO.**
    a. How many WA Medicaid lives do you cover?
    b. What other markets are you in besides WA Medicaid?
    c. How many regions is your MCO in and for how long have you been in those regions?

Now that we know that you have experience in X regions, we would like to hear more generally about your experiences transitioning to IMC.

3. **Can you tell me about your experience with the transition to IMC?**
    a. What were the key activities that needed to be done in order to transition?
    b. What challenges did you encounter?
        a. [What about reimbursement delays?]
    c. What strengths or supportive factors influenced your experience?

4. **How did your experience vary by region?**
    a. Probe for differences in each region [bold which regions apply to current interviewee]: Southwest, North Central, North Sound, Greater Columbia, King County, Pierce County, Spokane, Great Rivers, Thurston Mason, Salish
    b. How do you mentally organize or group each of the regions?
    c. Who did you typically work with?
    d. How was the work generally organized?

© 2023 McConnell KJ et al. JAMA Health Forum.

e. What set-ups were most helpful? (e.g., workgroups to help with integration roll-out, infrastructure for coordinating transition)

I'd love to better understand how payment has changed with the transition to IMC.

5. Can you tell me how mental health is now paid for in your region?
   a. Does the MCO hold full risk for the behavioral health benefit and manage the benefit, or do they subdelegate that to a county or counties or some other entity?
   b. Are there specific networks or models of care that are intended to serve individuals with serious mental illness**?**
   c. Can you tell me how SUD services are now paid for in your region?
   d. How is this different from before IMC?

We have heard about some difficulties with claims rejections and reimbursement delays. We understand that there are multiple sides to every story, and we want to ensure we understand this from your perspective.

6. Could you share your experiences with claims processing?
   a. What do you think contributed to this experience?

7. Please tell me about your MCO's experience with contracting with new provider organizations.
   a. What priorities did your MCO consider when contracting with new providers?
   b. What else influenced these new contracts you developed?
   c. To what extent did your experience contracting with providers vary by region?
      a. What factors do you think influenced this variation?

We understand this transition took a lot of work.

8. Could you describe for me what aspects of IMC integration were accomplished before the IMC deadline in your regions?
   a. What work remained after the deadline?
   b. What work still remains?

Now I'd like to ask you about the impact of the Integrated Managed Care transition.

9. What have been the impacts of integrated managed care?
   a. What impact has IMC had on integration at the care delivery level?
   b. What impact has IMC had on patients?
   c. What impact has IMC had on primary care practices?
      a. Has their ability to pay for certain services changed?
   d. In what ways does IMC foster clinical integration? How so?
   e. What else is needed for provider organizations to integrate care at the care delivery level?

10. What patient populations have been impacted by the transition to IMC?
- In what ways?
- Adults vs. children?
- Can you tell me about how IMC influenced care for individuals with serious mental illness? How does this compare to those with mild or moderate mental health conditions?

© 2023 McConnell KJ et al. JAMA Health Forum.

This is new and important work, and there may be other states that follow in Washington's path.

11. What lessons from your experience with IMC integration in Washington would you share with people in other regions or states?

- What resources are needed?
- What supports should be available?
- What types of data-sharing abilities are required?
- What types of workforce capacity issues should be considered?
- What should states know about the intersection of Value-Based Payment and Integrated Managed Care?
- State role/leadership?
    - What could the state have done differently to improve this process?
- [optional] You've worked in so many regions. By the time you get to region six you may have developed a sense of what contributes to a successful transition. What are the key components that contribute to a smooth IMC transition?

12. Before we close, what have we not asked about, but should know, about your experience related to integrated managed care?

13. To better understand the IMC transition across Washington state, who else would you recommend we speak to?

© 2023 McConnell KJ et al. JAMA Health Forum.

eMETHODS 2

Qualitative methods

Qualitative Data Collection and Analysis

Interviews were conducted between October 2021 and April 2023 using a semi-structured guide. State and regional leaders helped us identify individuals who were knowledgeable about IMC. We purposively selected 24 participants from those referrals to ensure we interviewed people representing all regions and managed care organizations (MCOs) and with varied background and viewpoints. We utilized member checking, wherein data was returned to select participants to check for accuracy and resonance of their experiences with IMC. Participants included individuals who facilitated the transition to IMC in their region, leaders at behavioral health agencies, and MCO administrators.

Participants were provided an information sheet that outlined the purpose of the study, participation risks and benefits, and processes for deidentification. We contacted study participants to request permission to publish any quotes included in the manuscript. Interviews averaged an hour, were conducted by video or over the phone, audio-recorded, professionally transcribed, and deidentified. Transcripts were entered into Atlas.ti for data management and analysis. We iteratively conducted data collection and analysis, using what we learned in early interviews to inform subsequent sampling and interview guide questions and to monitor for saturation. A research team with expertise in qualitative methods and integrated care analyzed the data.

We used an inductive data coding approach. We developed the codebook iteratively, and for the first set of data, we coded transcripts as a team and tagged segments of text from the interview transcripts together. When the codes and definitions were clear and consistently used among the team, two authors (JH and MD) independently coded the remaining data, and met to discuss each transcript and to review the codebook. Discrepancies were analyzed and reconciled, and a new version of the codebook was prepared. We continued to add new codes and revise code definitions as we collected and analyzed more data.

Following coding, the team met to identify emerging preliminary findings and implications from the analysis using immersion crystallization methods.[22] During this period of reading and rereading, the team identified several factors that influenced the impact that IMC had on Washington practices and their ability to integrate

© 2023 McConnell KJ et al. JAMA Health Forum.

care. These were shared with the larger team, discussed during mixed methods meetings alongside the quantitative data, and further refined to identify the factors that influenced the impact of IMC on primary care practices (and individuals with mild-to moderate mental health needs) and behavioral health agencies that primarily served SMI populations.

© 2023 McConnell KJ et al. JAMA Health Forum.

eAPPENDIX 3– Event
Study Graphs

© 2023 McConnell KJ et al. JAMA Health Forum.

Detrended, Groups 2-5 Models

SMI

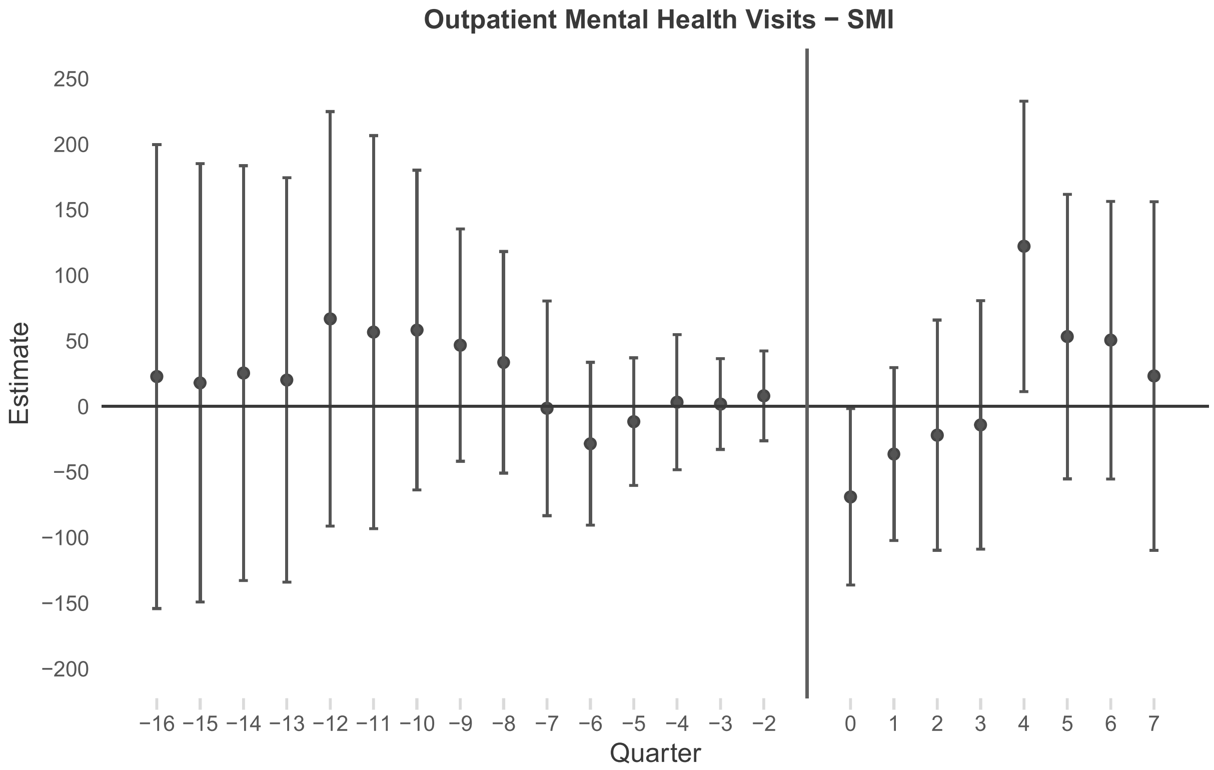

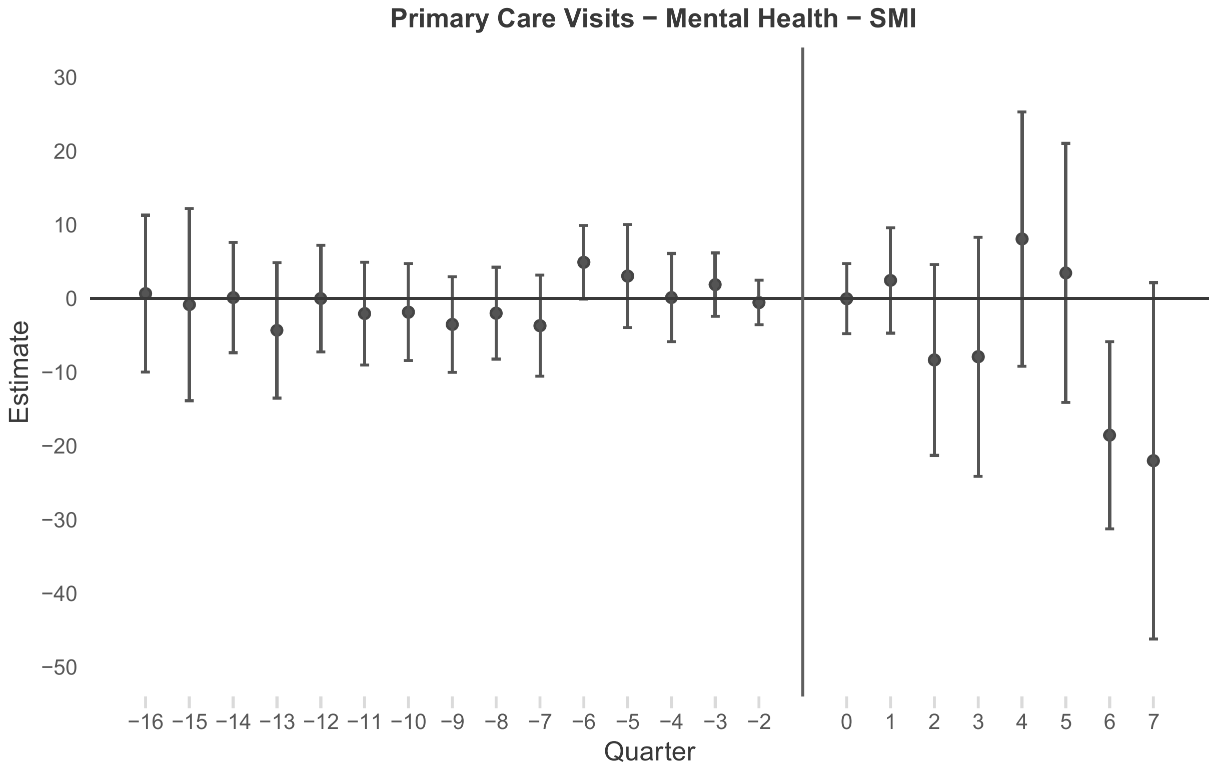

© 2023 McConnell KJ et al. JAMA Health Forum.

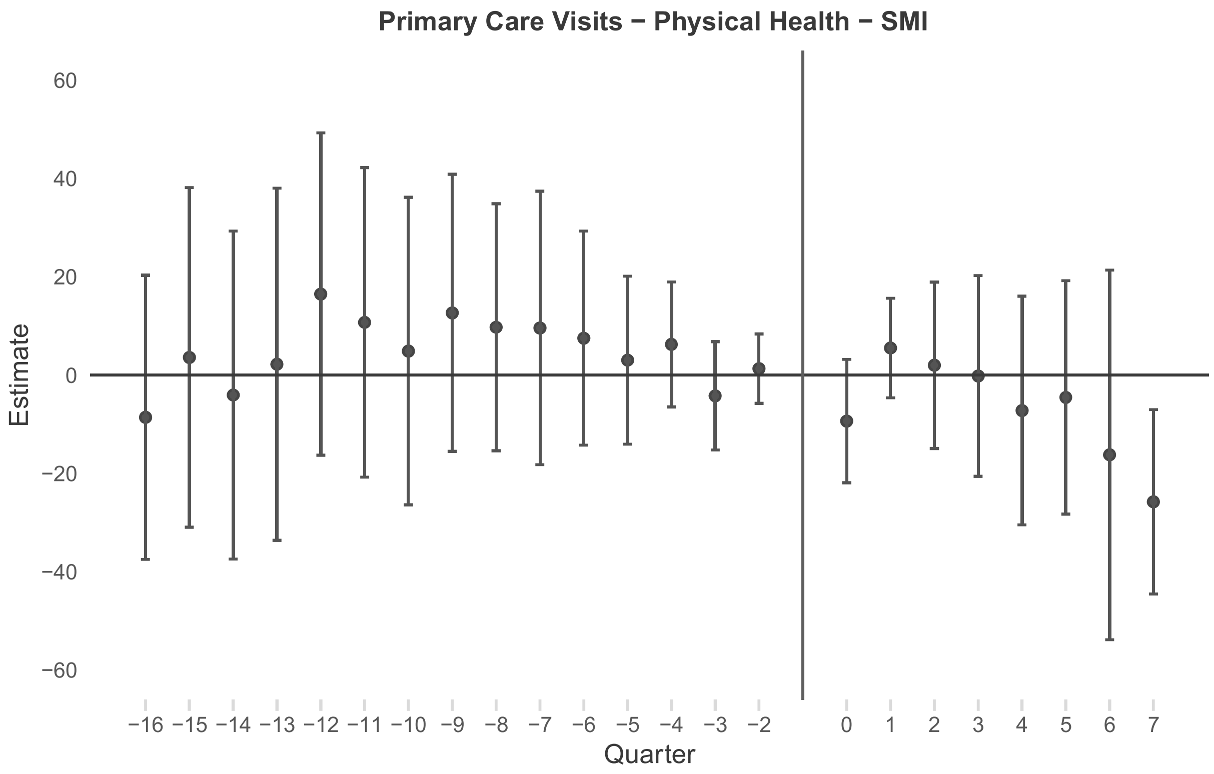

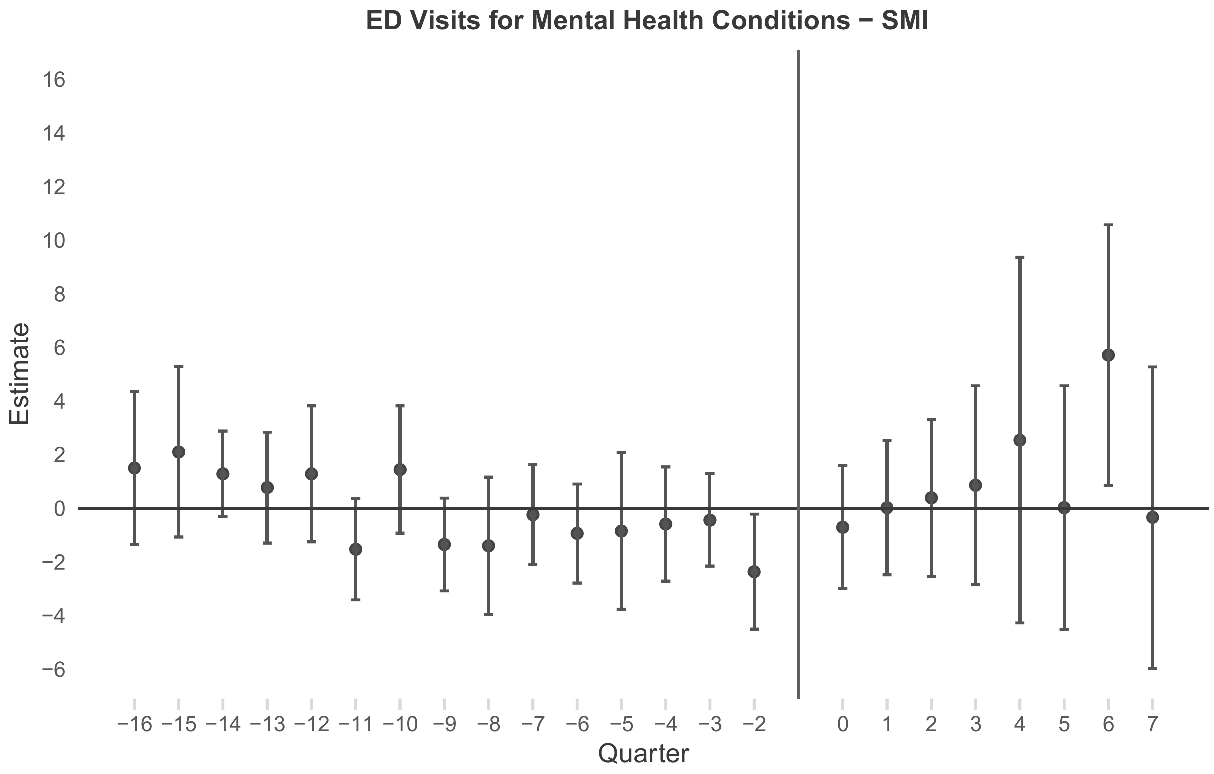

© 2023 McConnell KJ et al. JAMA Health Forum.

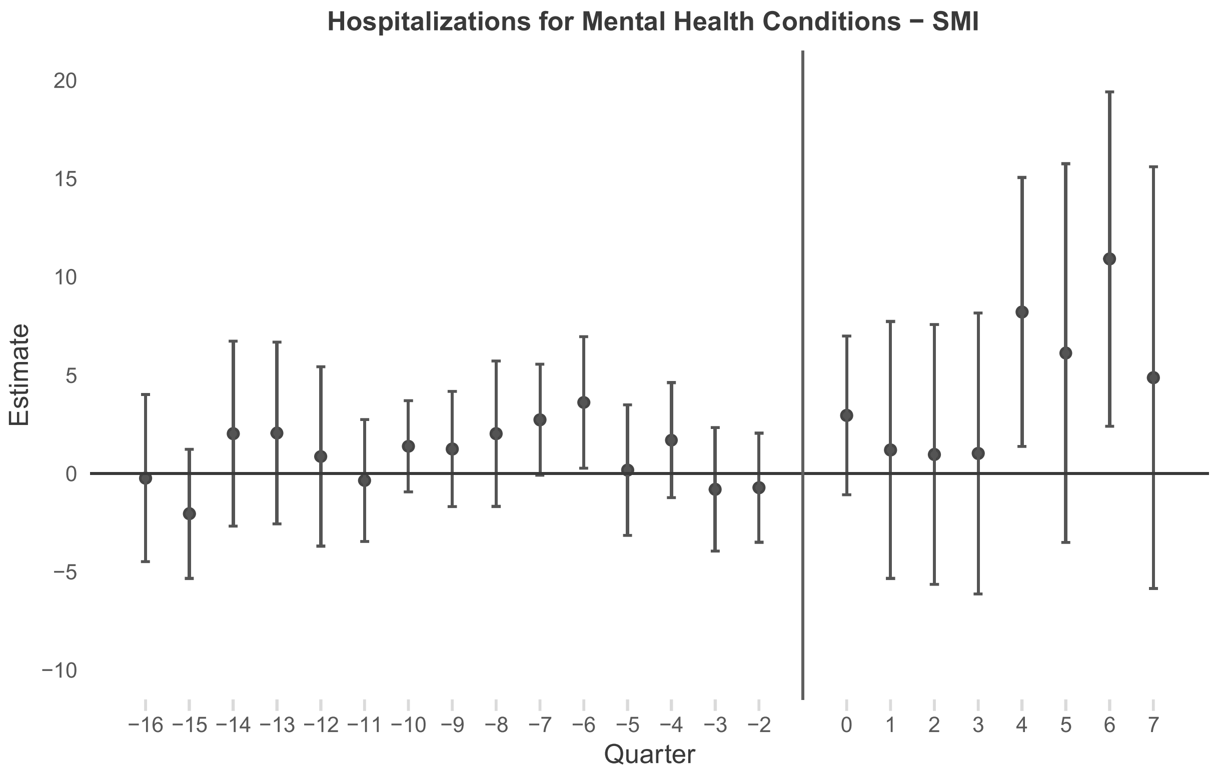

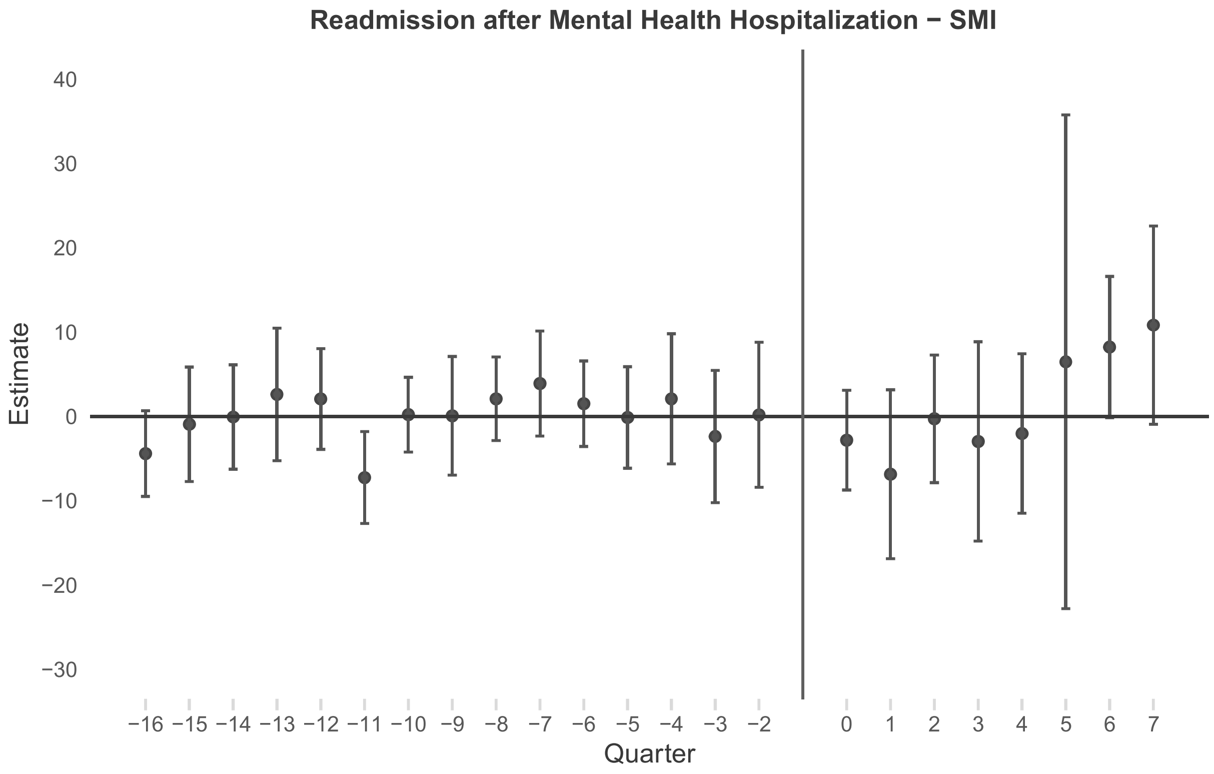

© 2023 McConnell KJ et al. JAMA Health Forum.

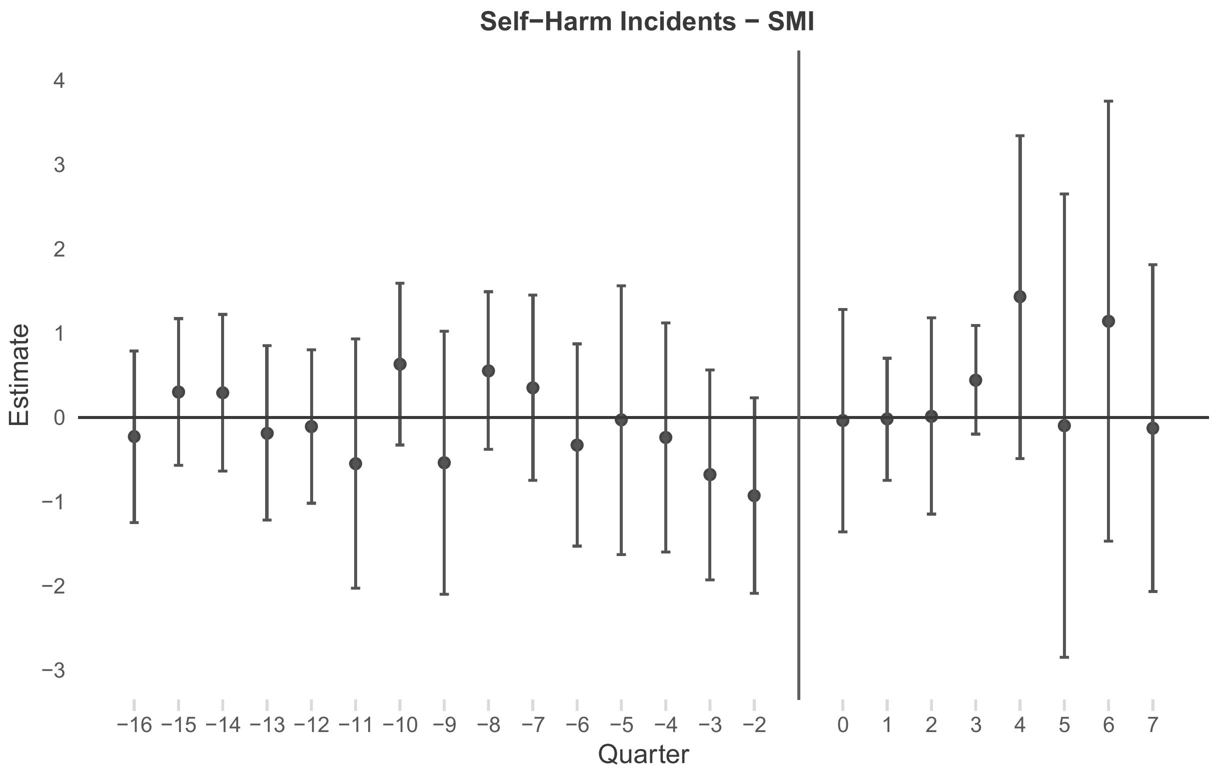

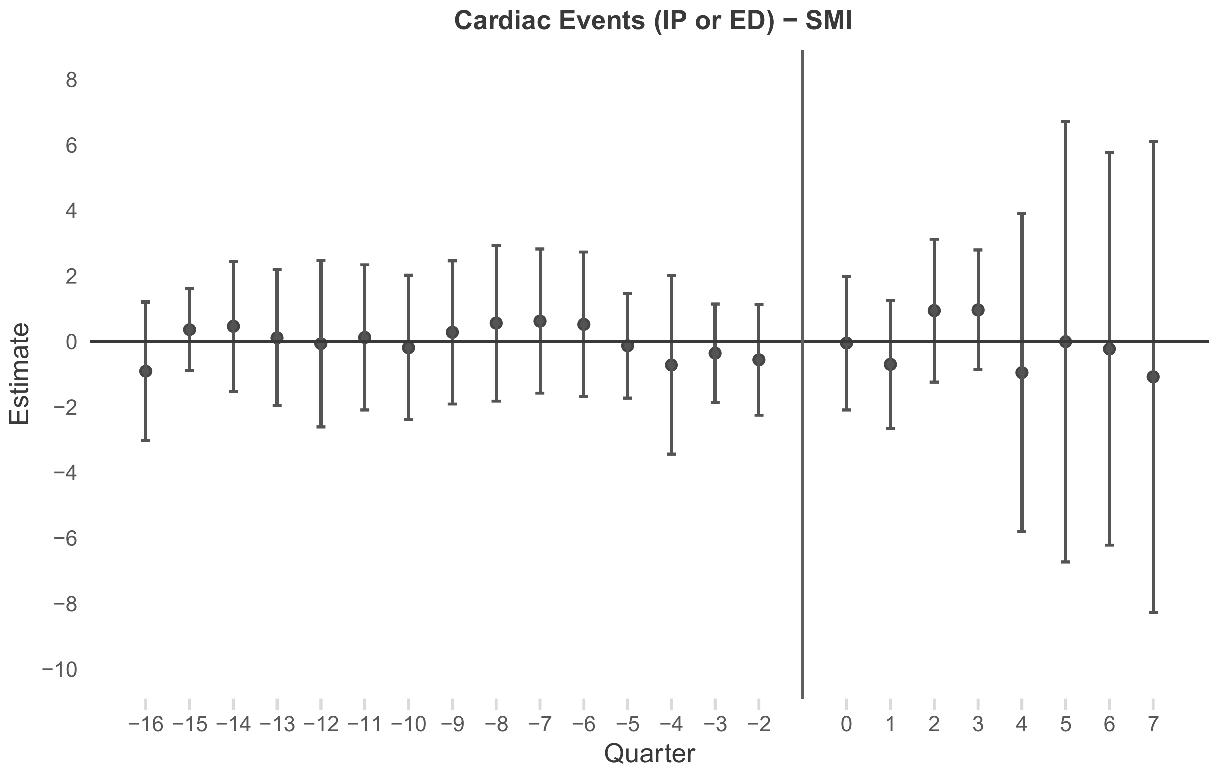

© 2023 McConnell KJ et al. JAMA Health Forum.

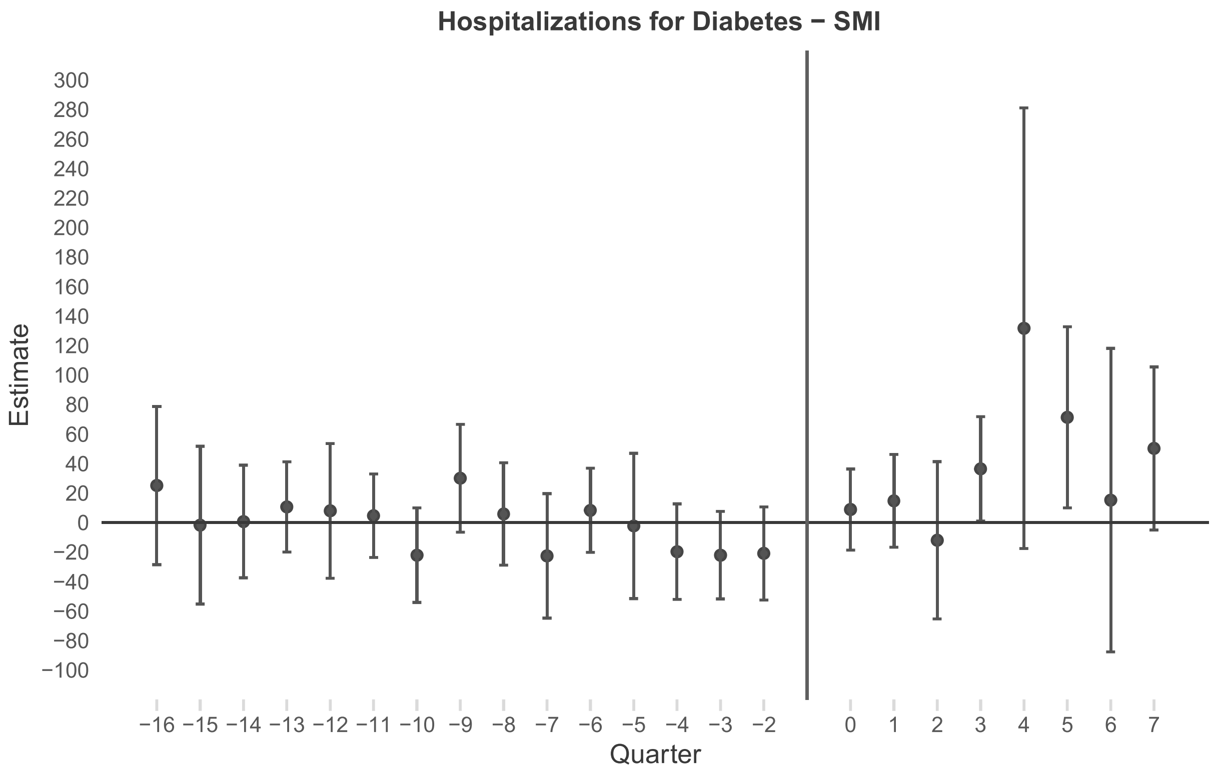

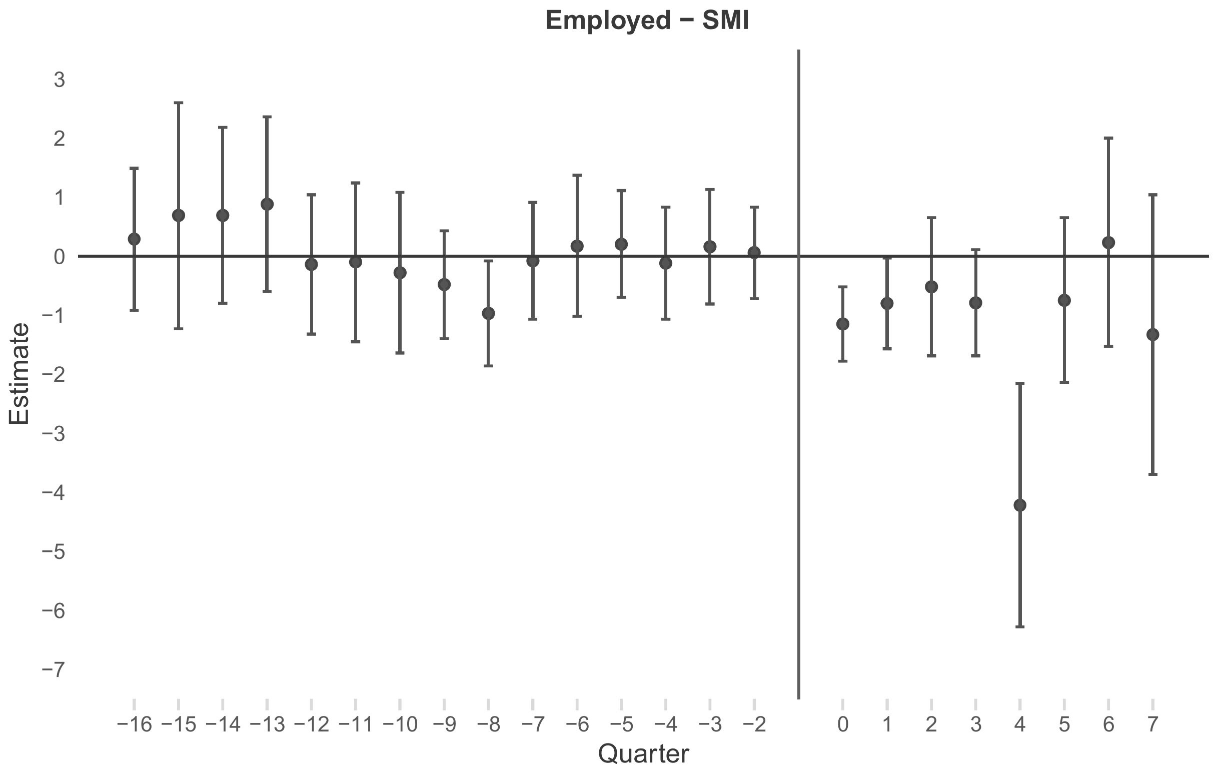

© 2023 McConnell KJ et al. JAMA Health Forum.

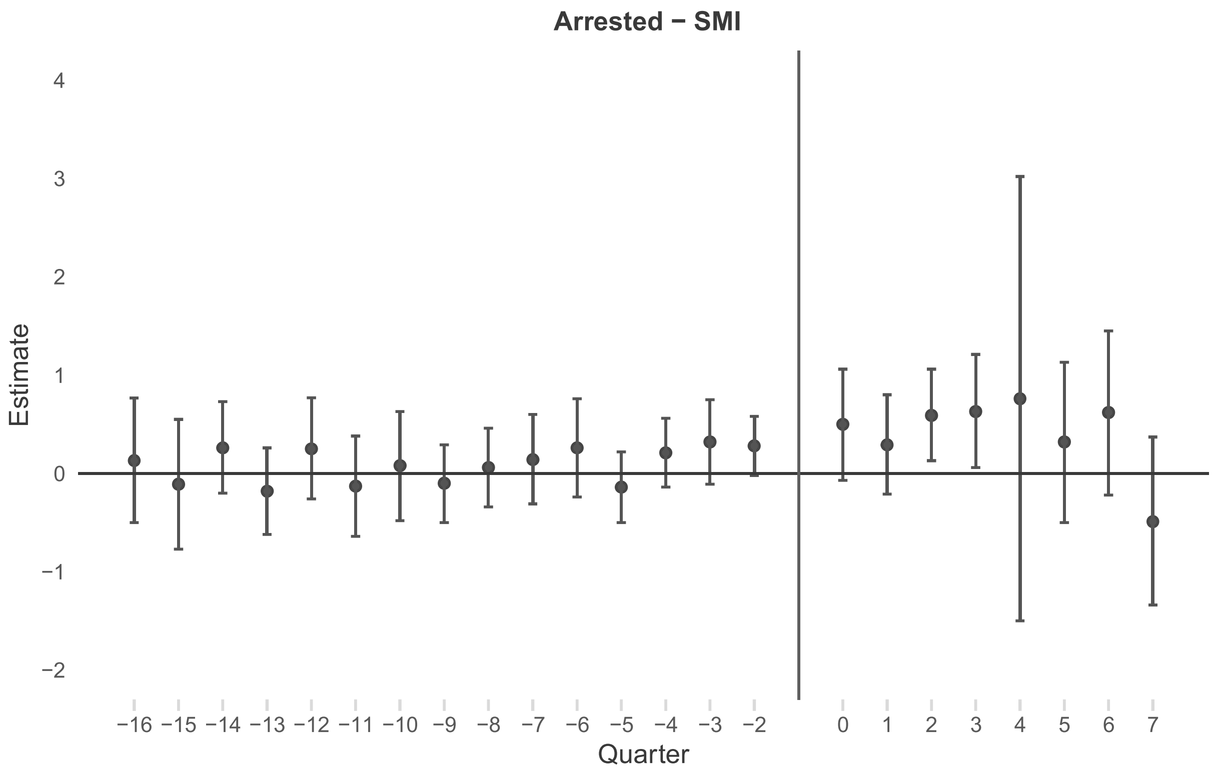

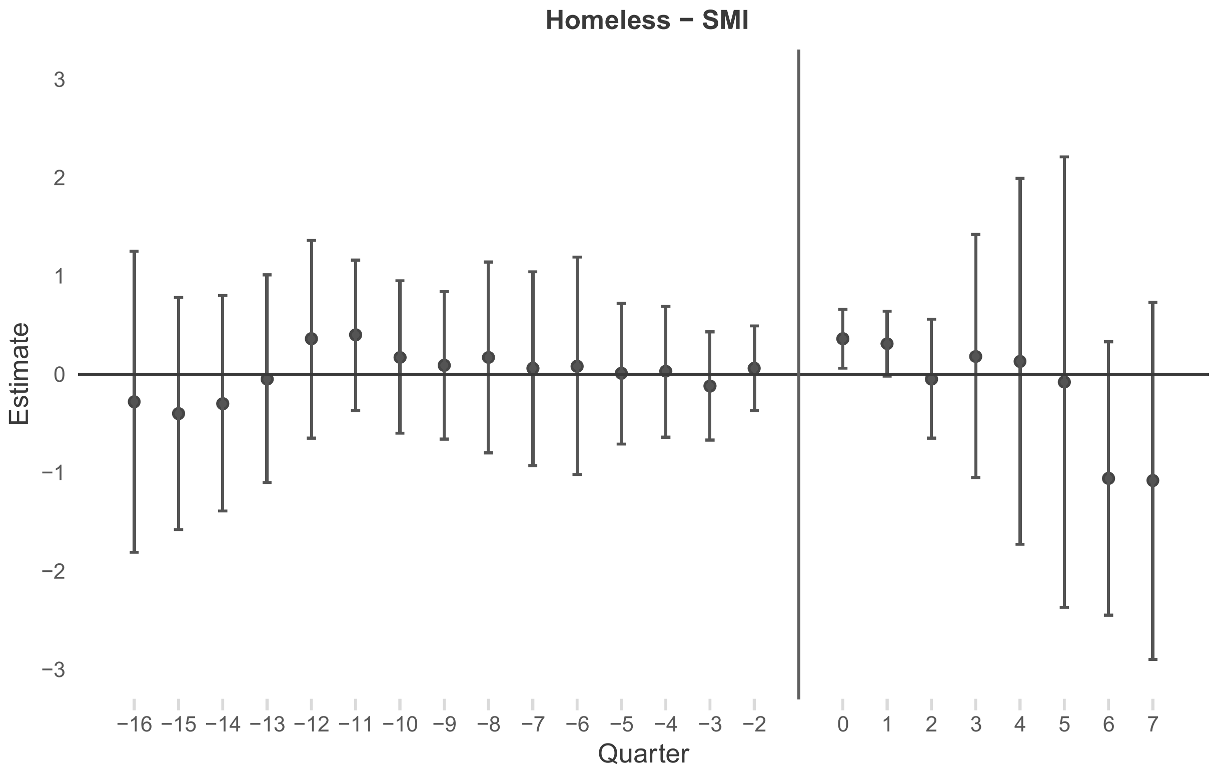

© 2023 McConnell KJ et al. JAMA Health Forum.

Mild-Moderate MI

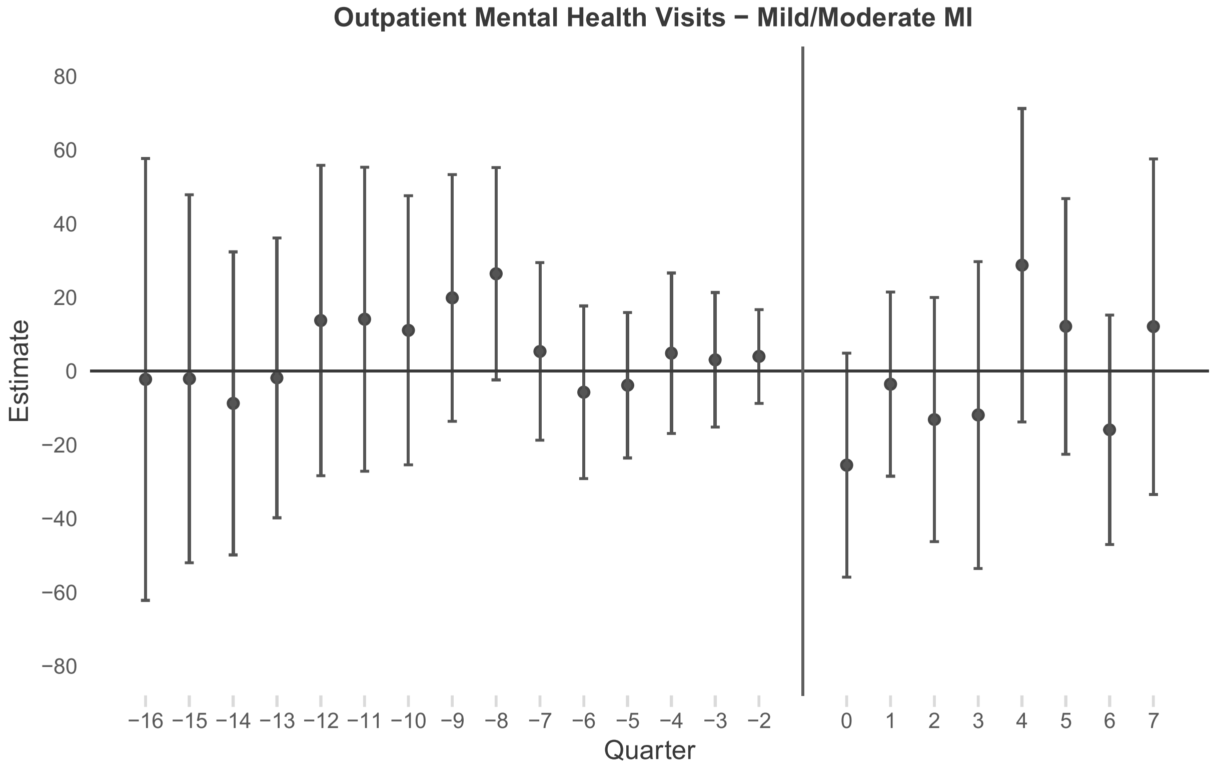

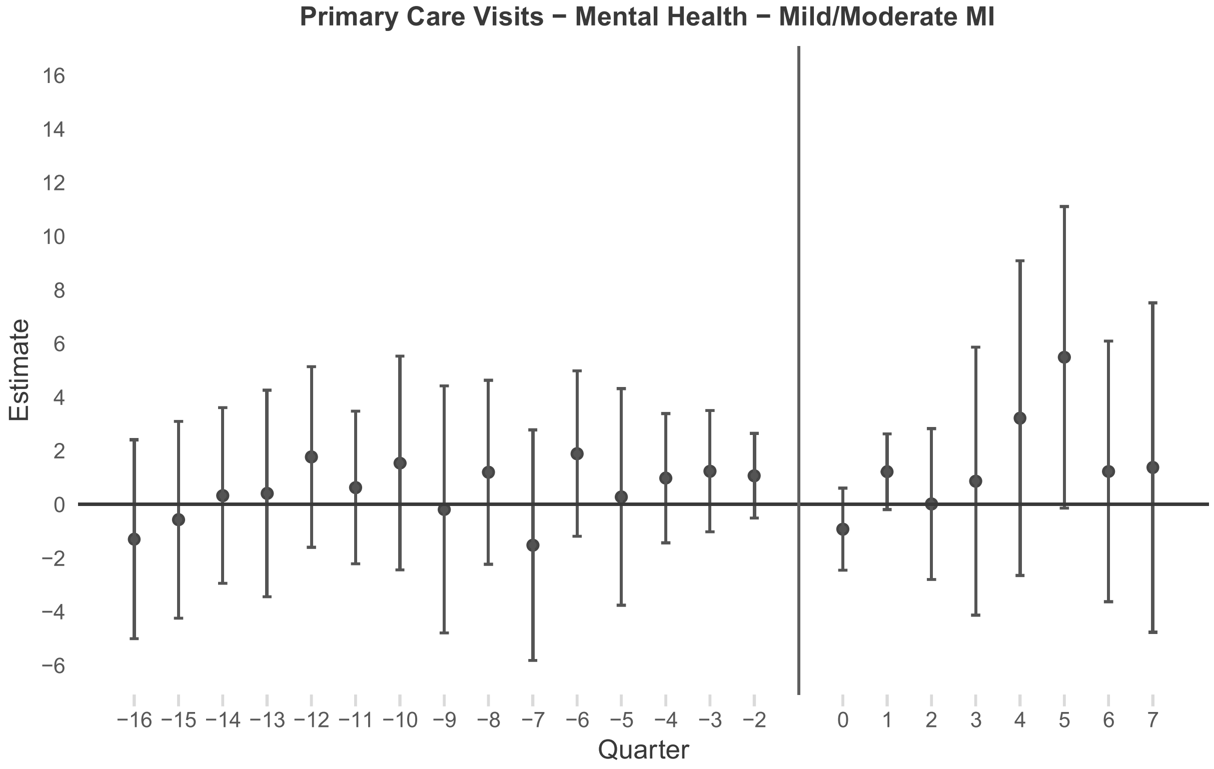

© 2023 McConnell KJ et al. JAMA Health Forum.

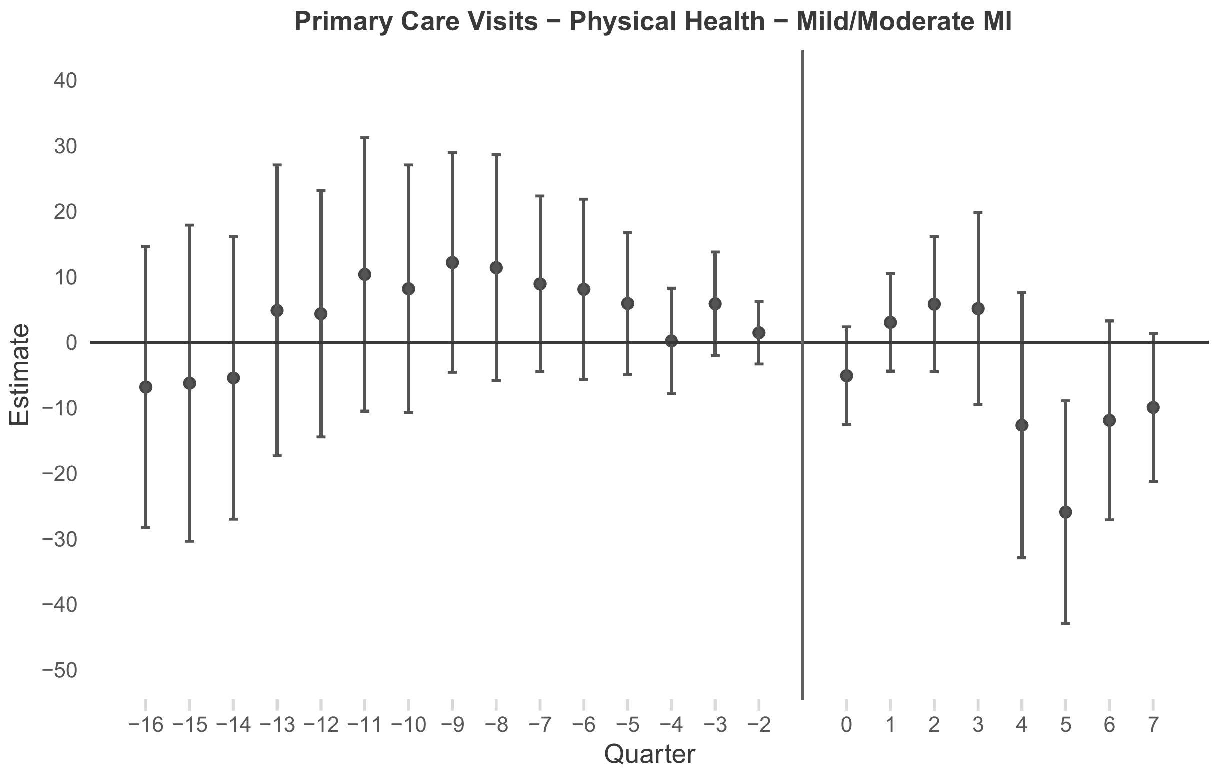

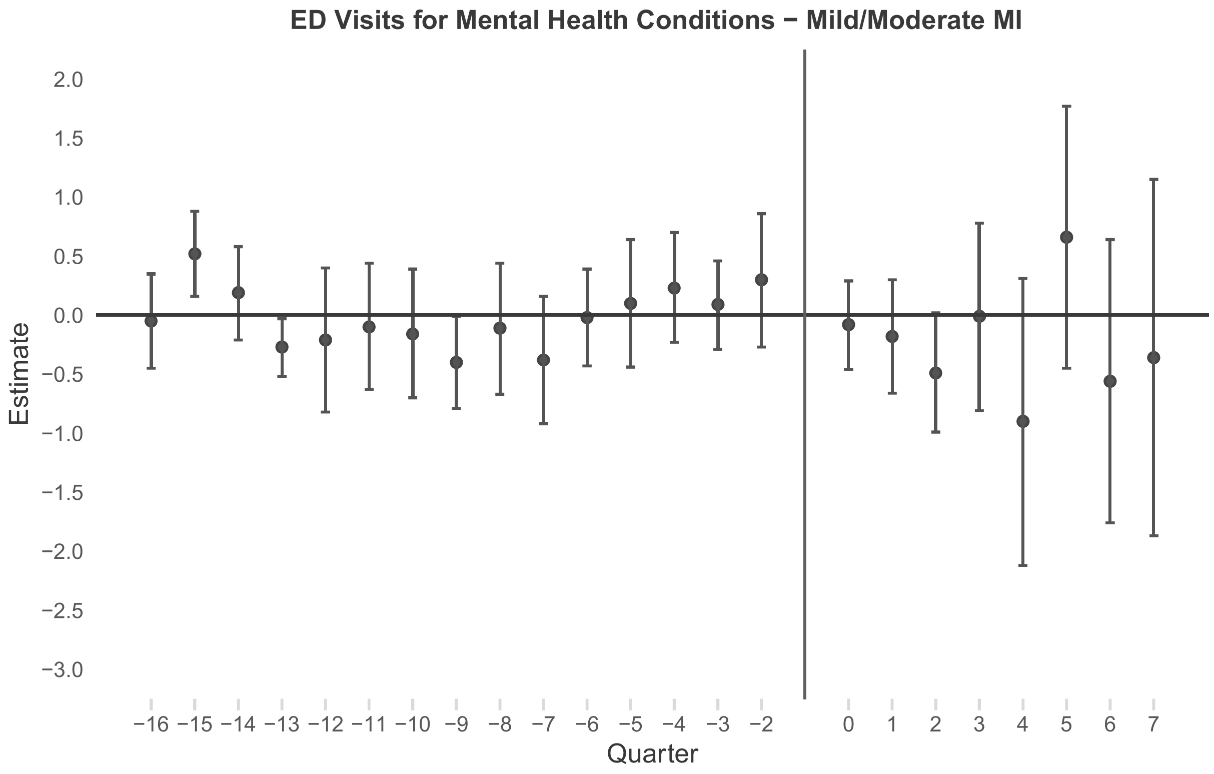

© 2023 McConnell KJ et al. JAMA Health Forum.

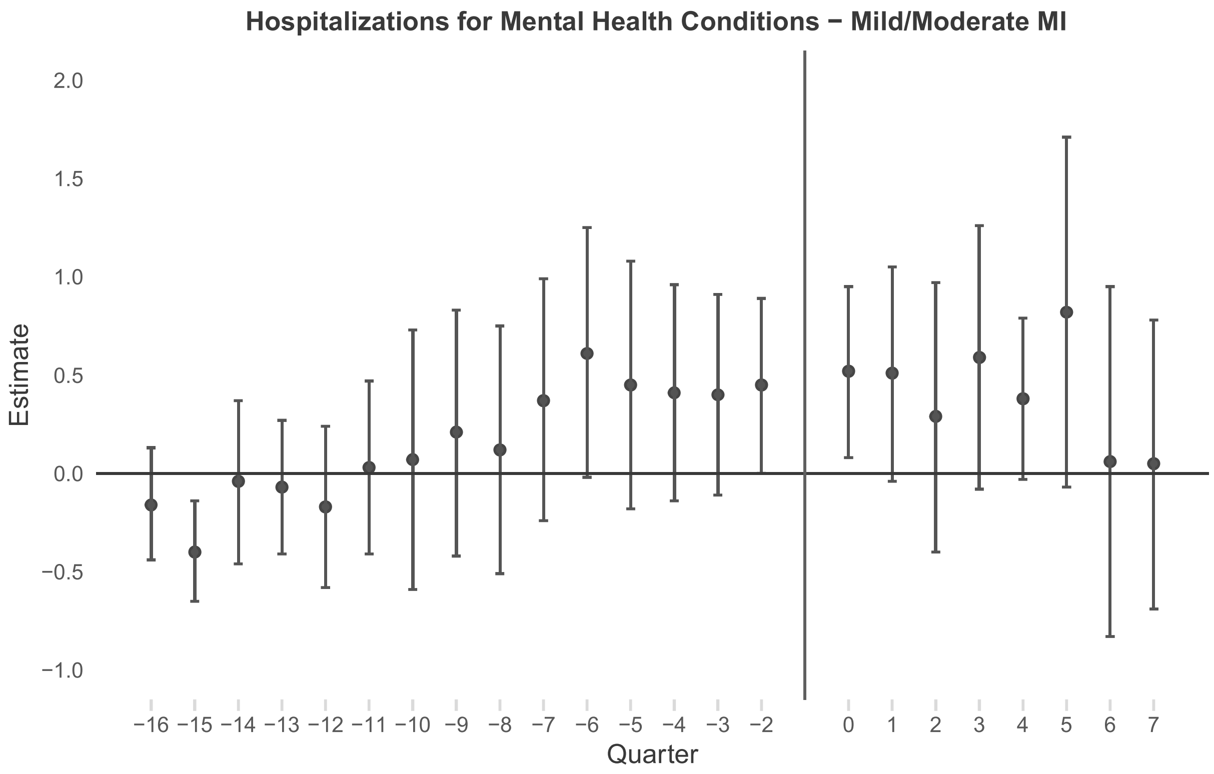

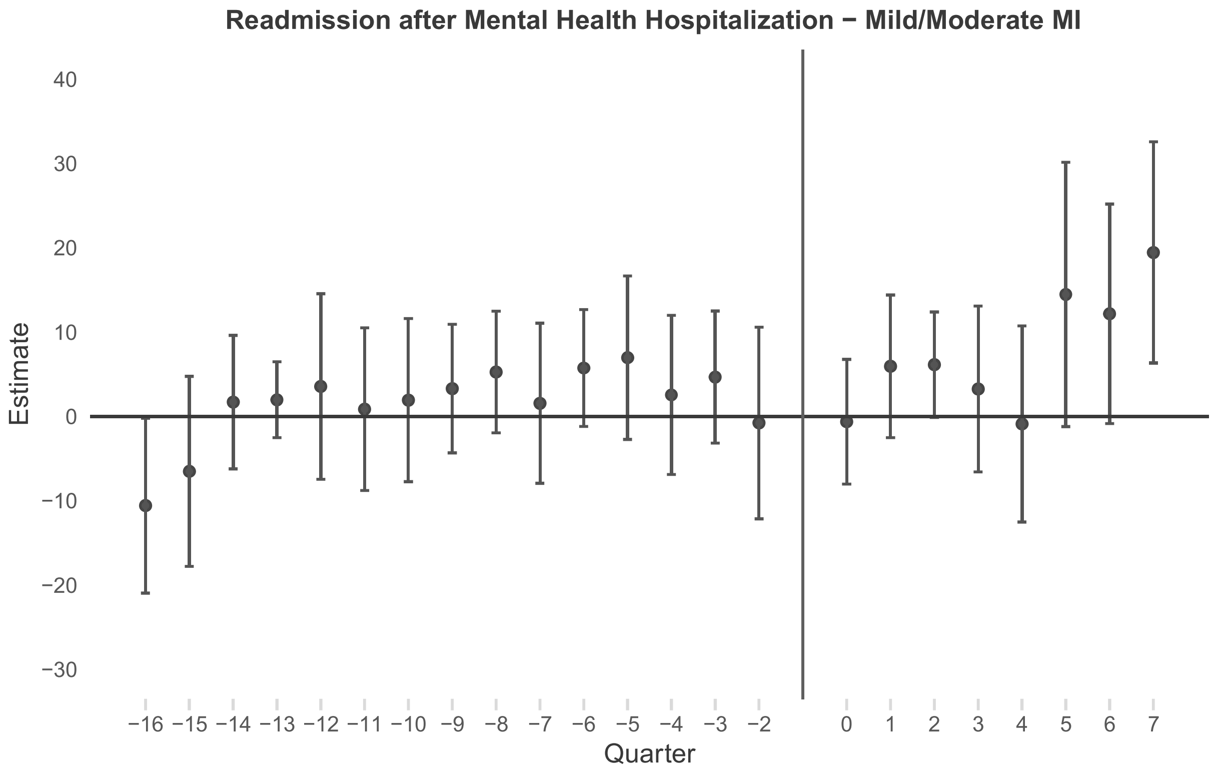

© 2023 McConnell KJ et al. JAMA Health Forum.

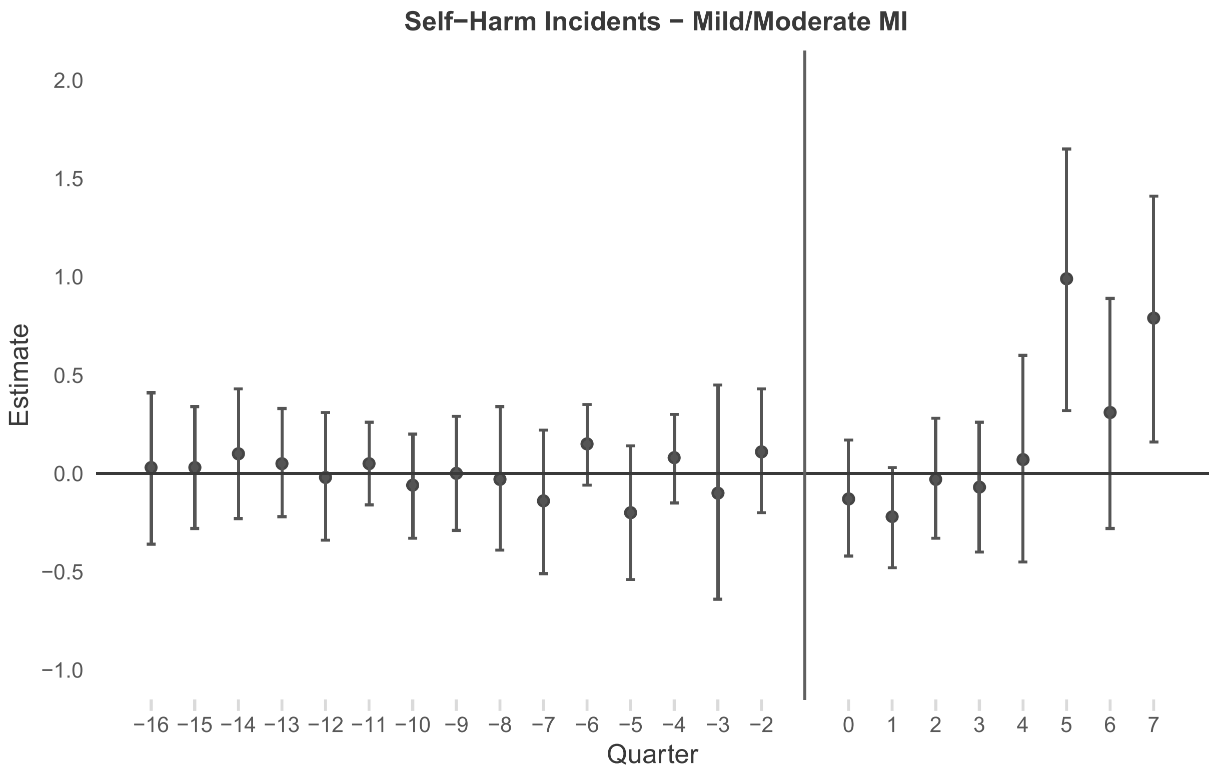

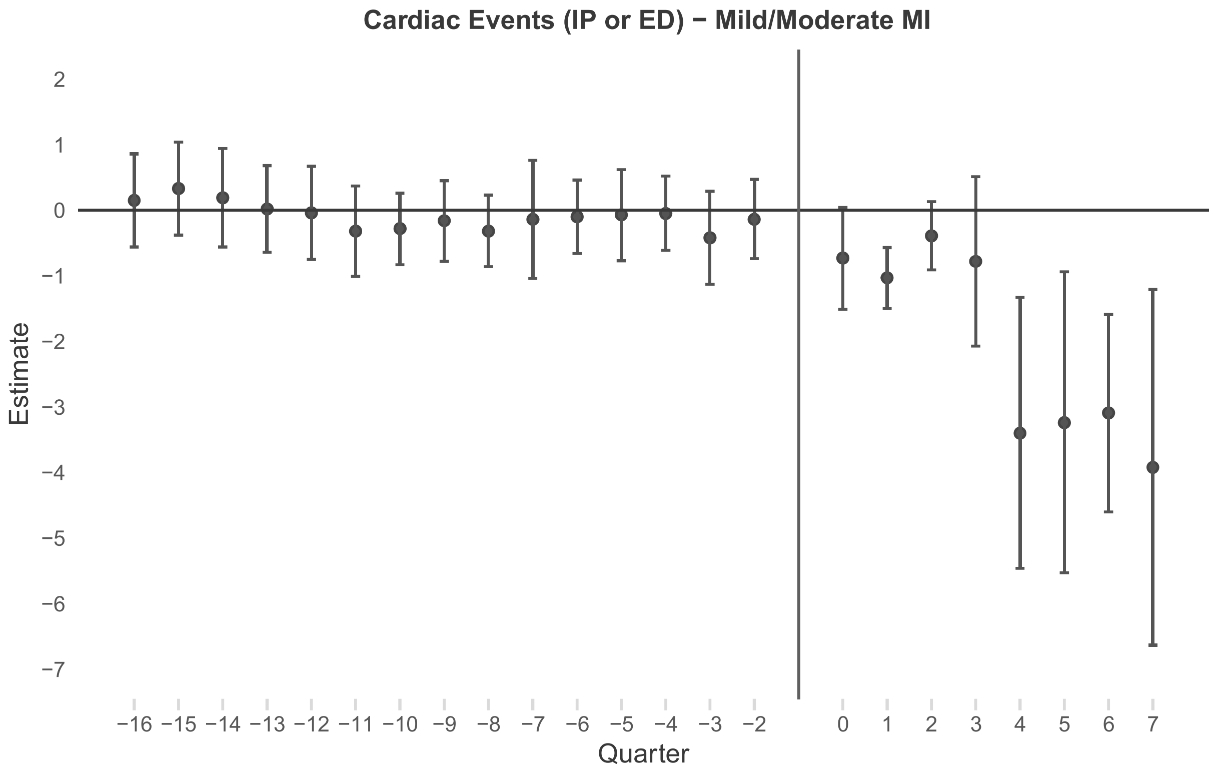

© 2023 McConnell KJ et al. JAMA Health Forum.

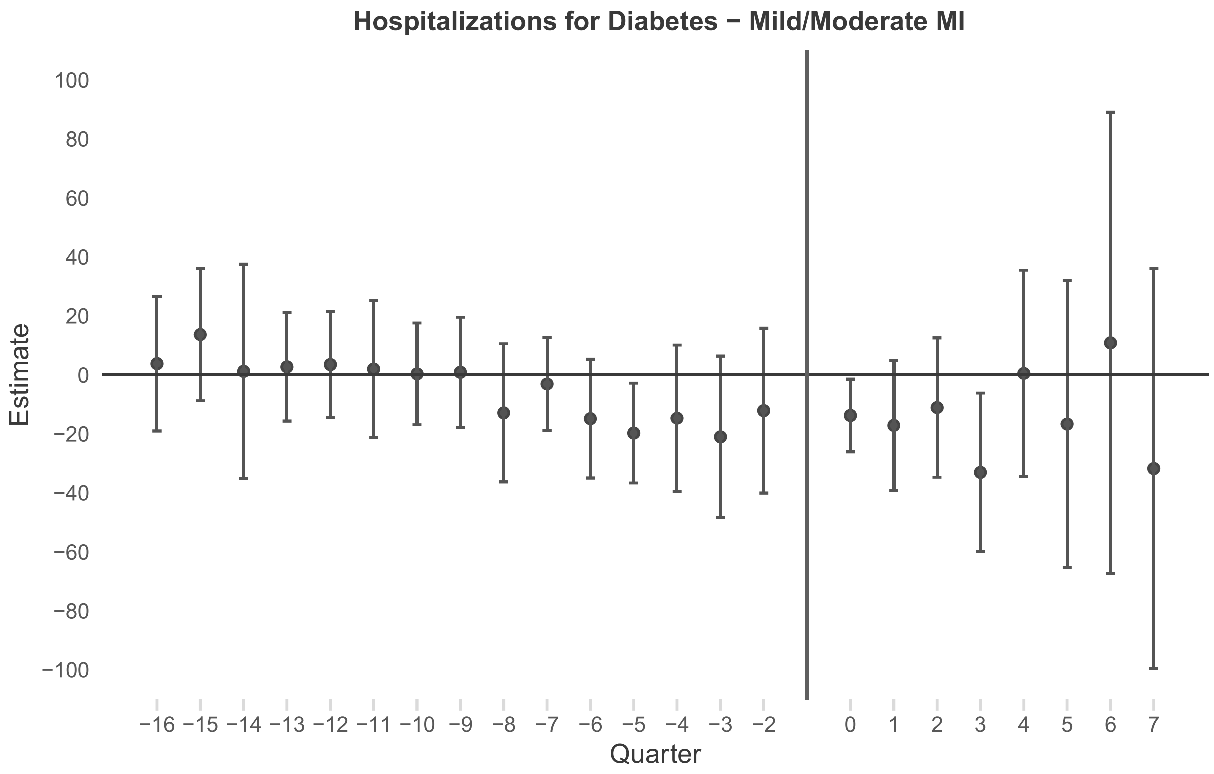

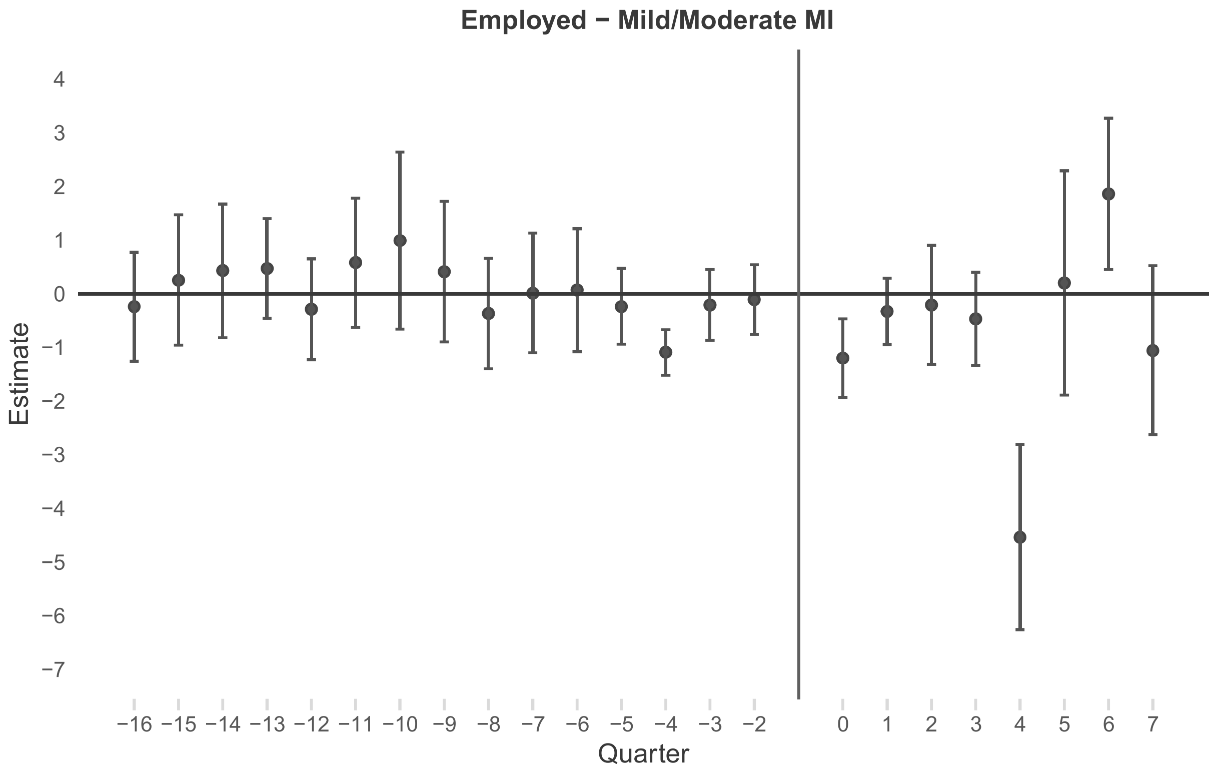

© 2023 McConnell KJ et al. JAMA Health Forum.

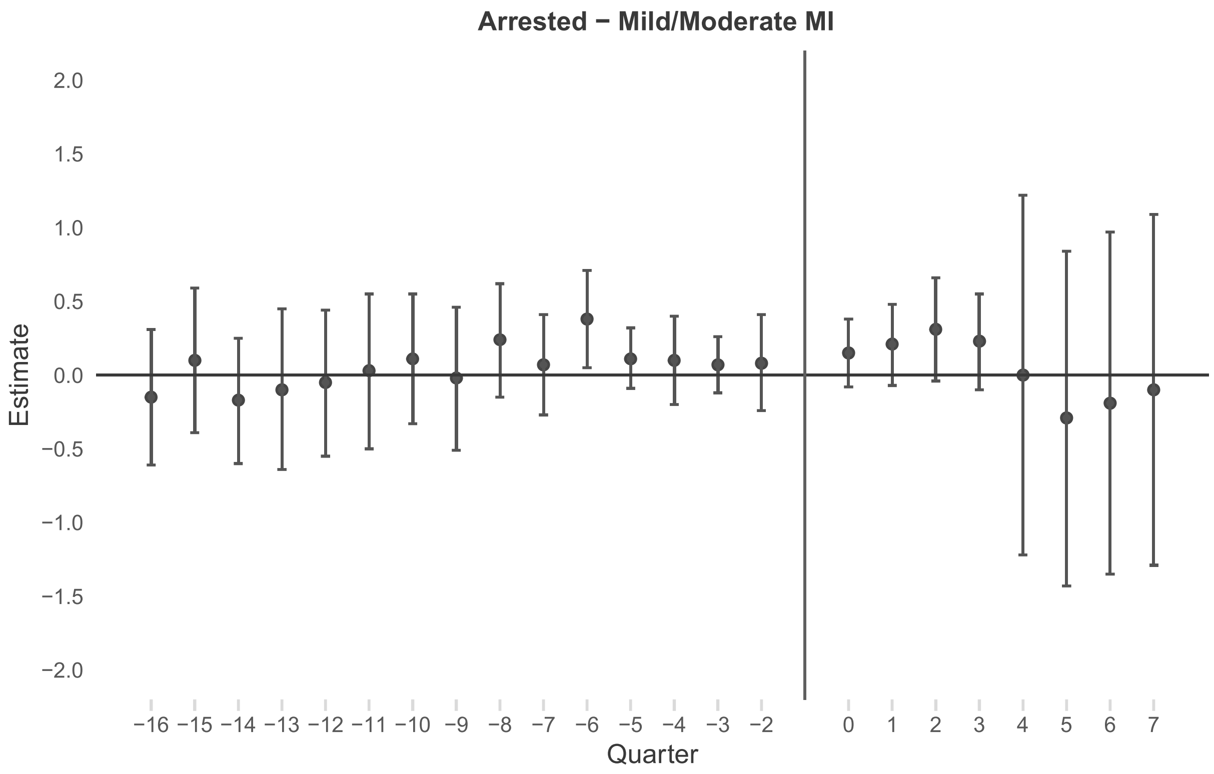

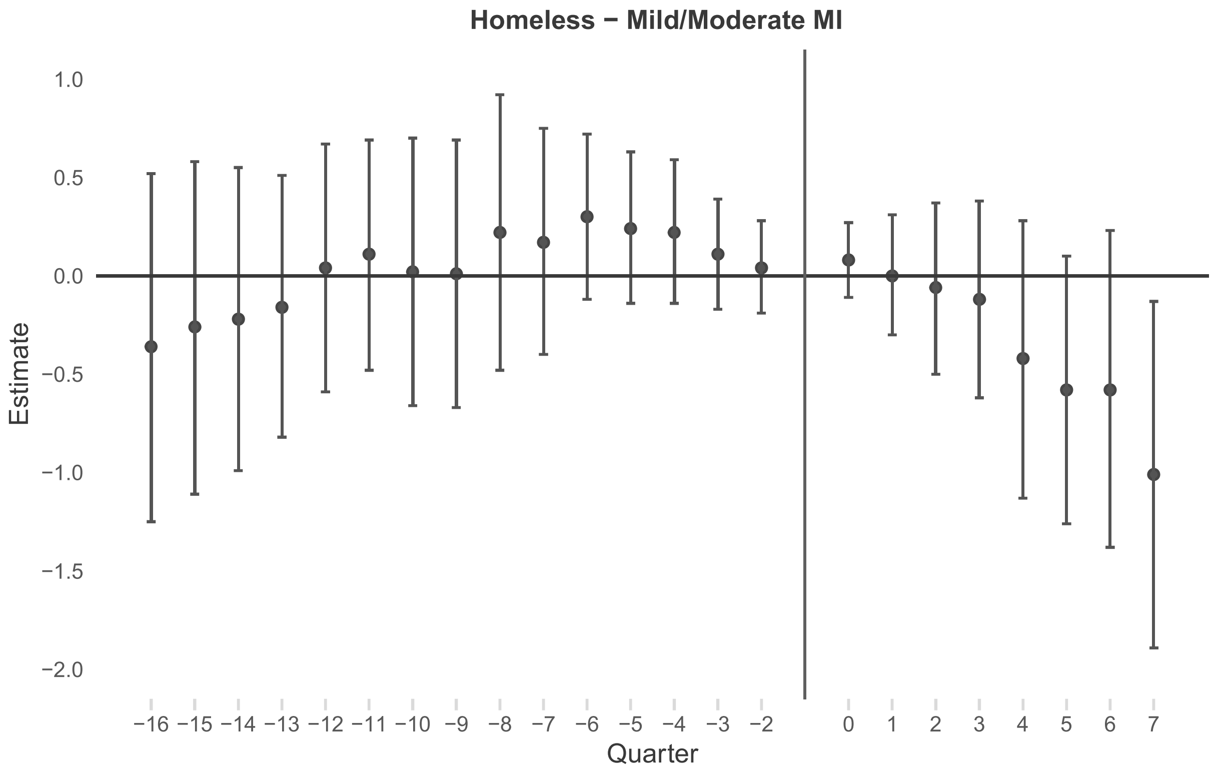

© 2023 McConnell KJ et al. JAMA Health Forum.

No MI

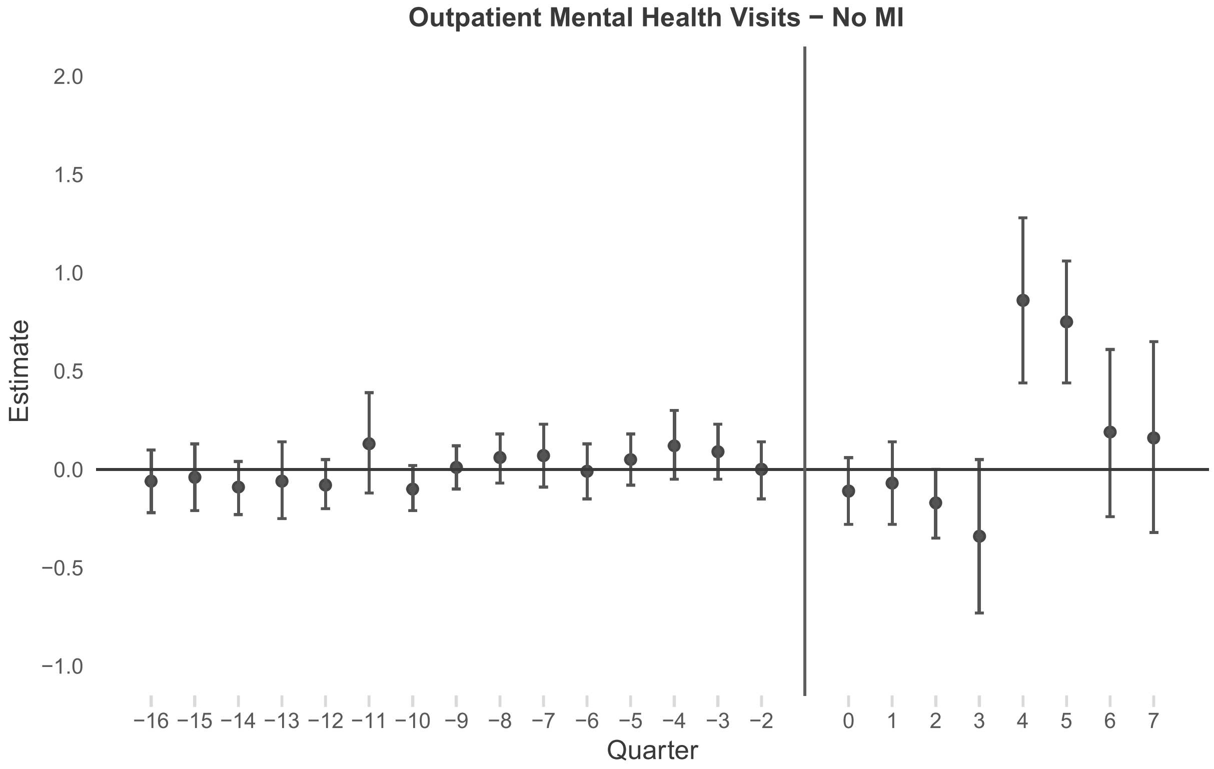

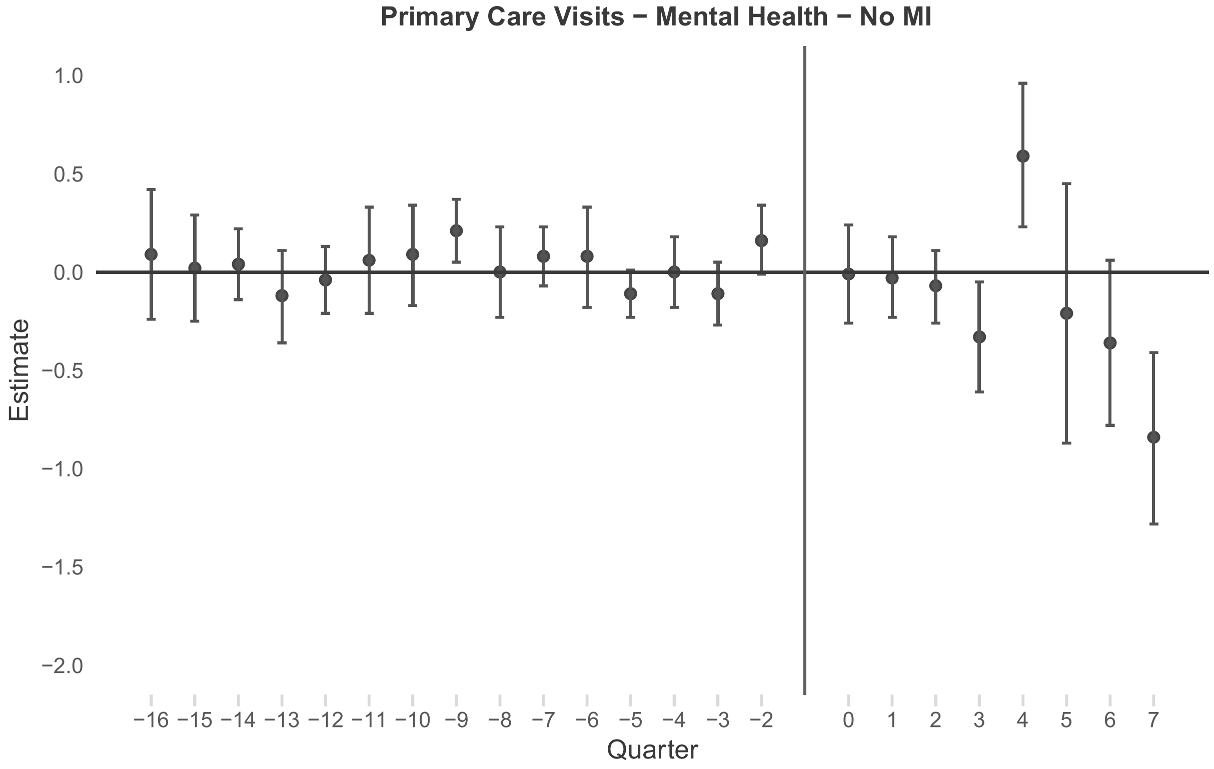

© 2023 McConnell KJ et al. JAMA Health Forum.

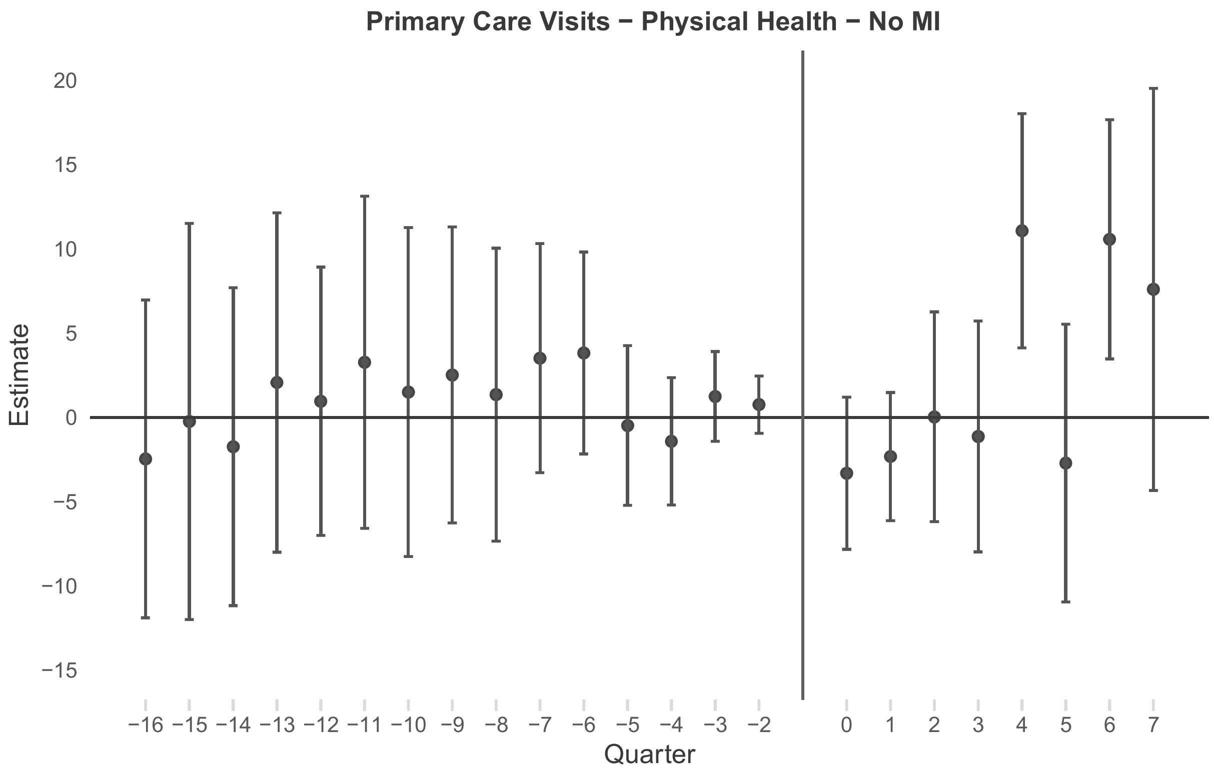

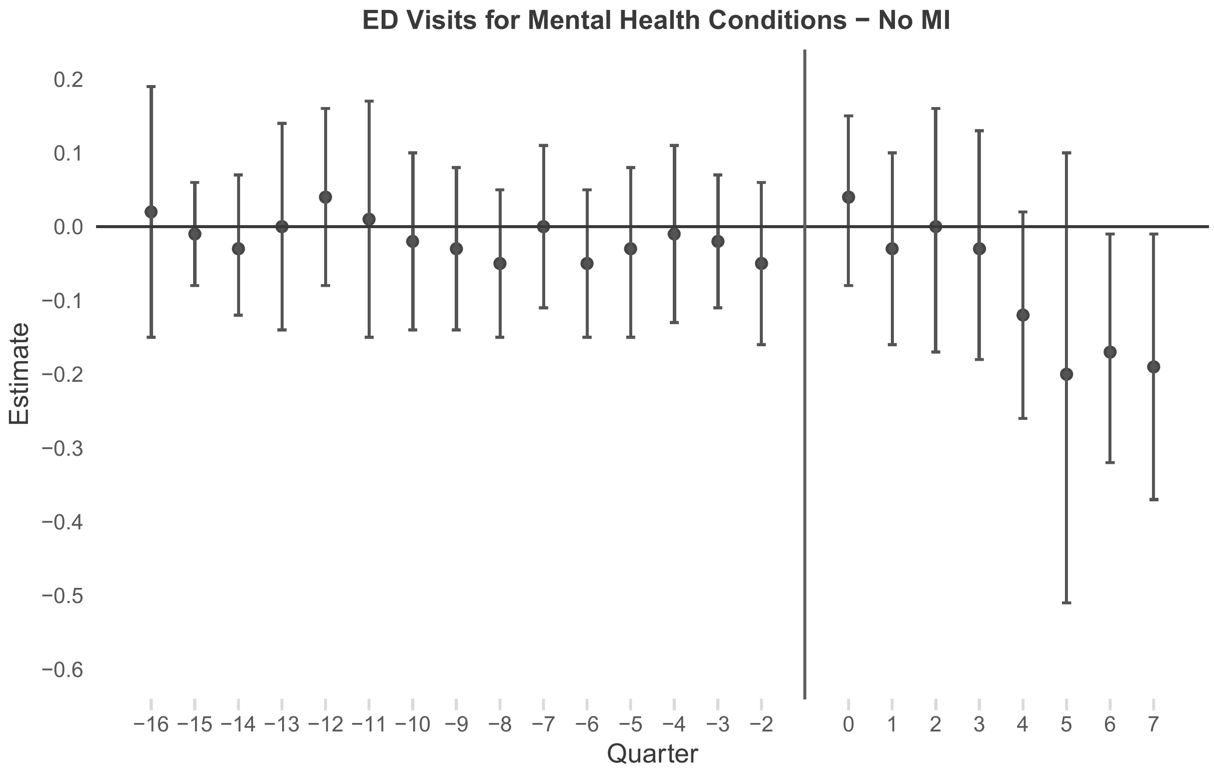

© 2023 McConnell KJ et al. JAMA Health Forum.

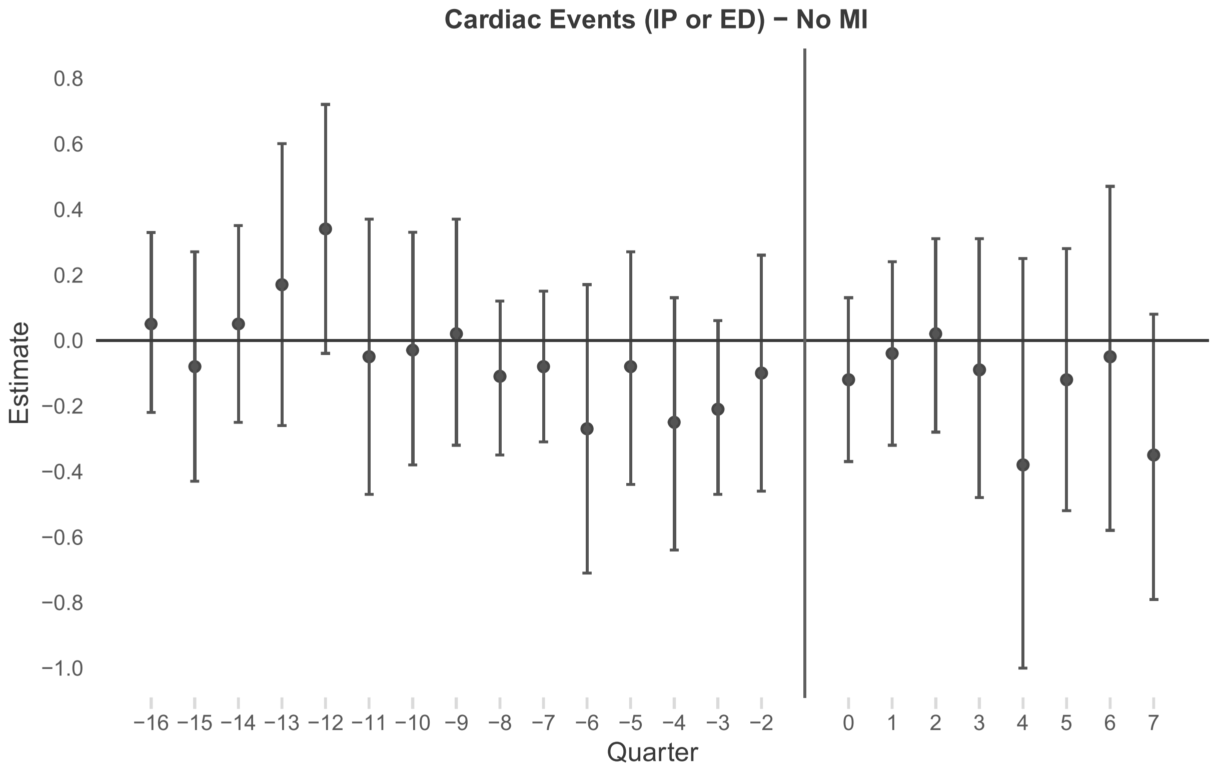

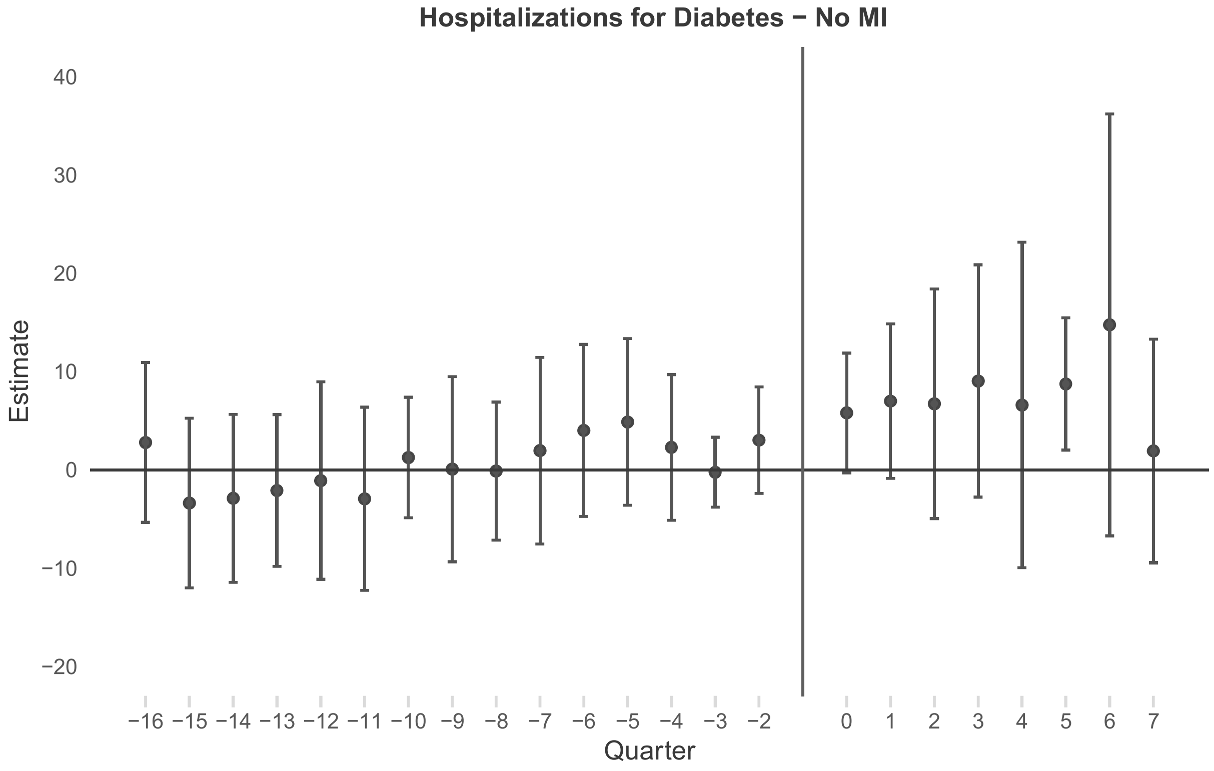

© 2023 McConnell KJ et al. JAMA Health Forum.

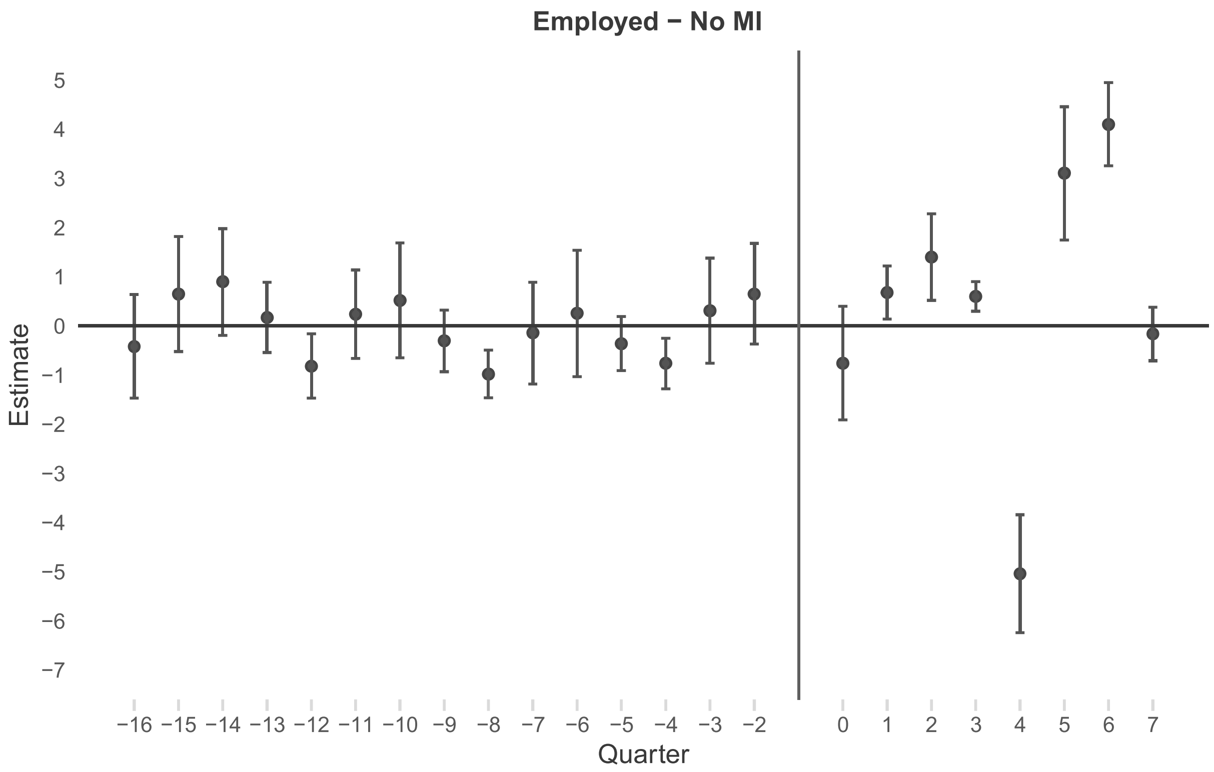

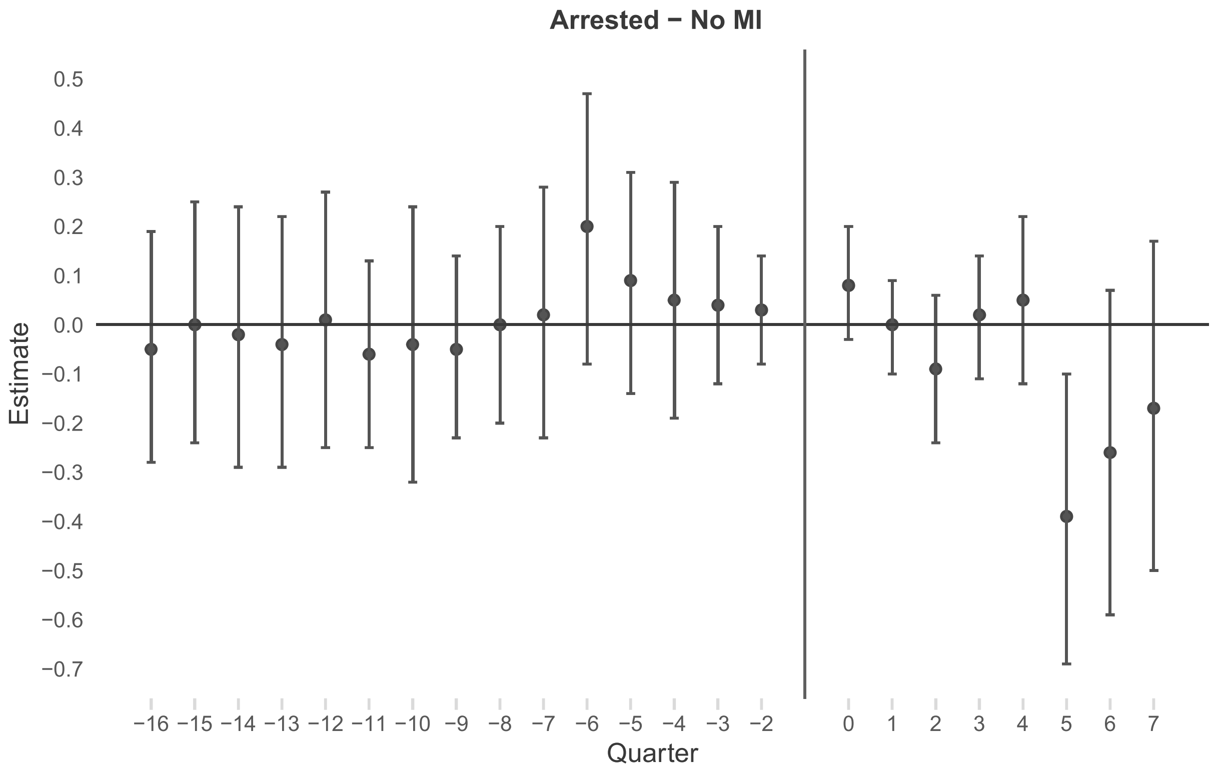

© 2023 McConnell KJ et al. JAMA Health Forum.

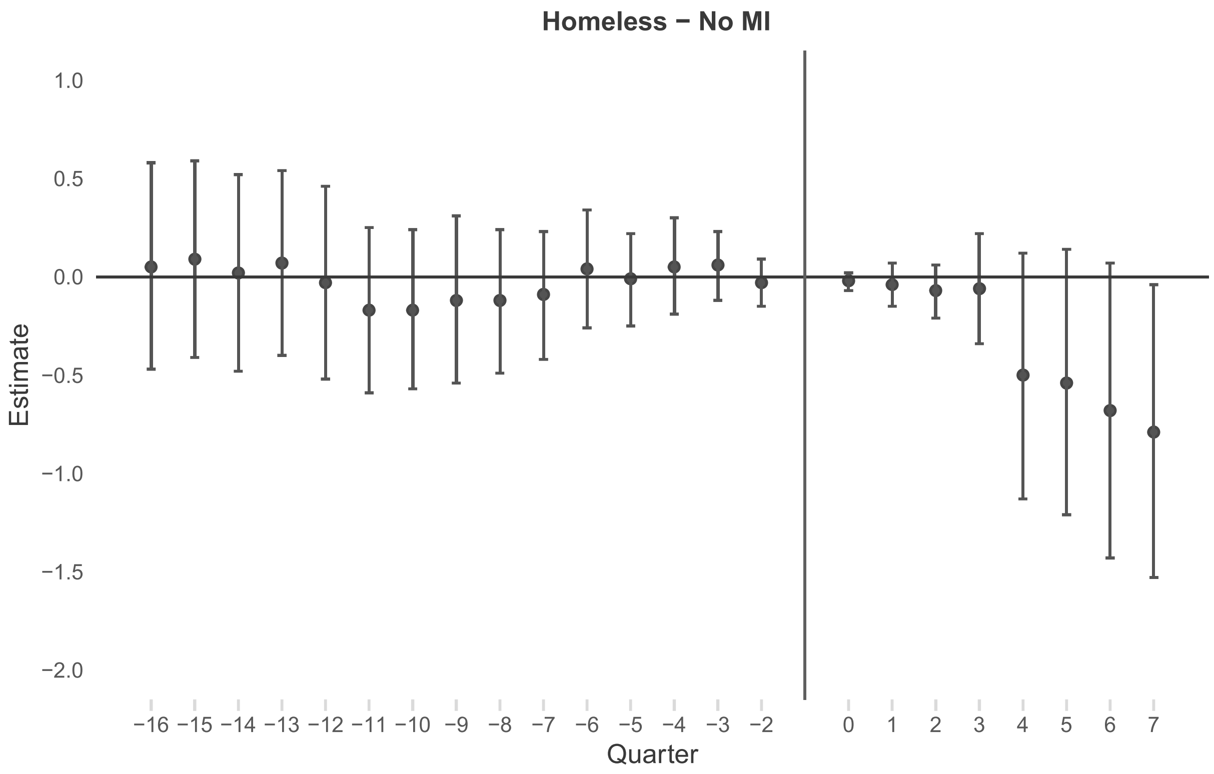

© 2023 McConnell KJ et al. JAMA Health Forum.

HEDIS Models

SMI

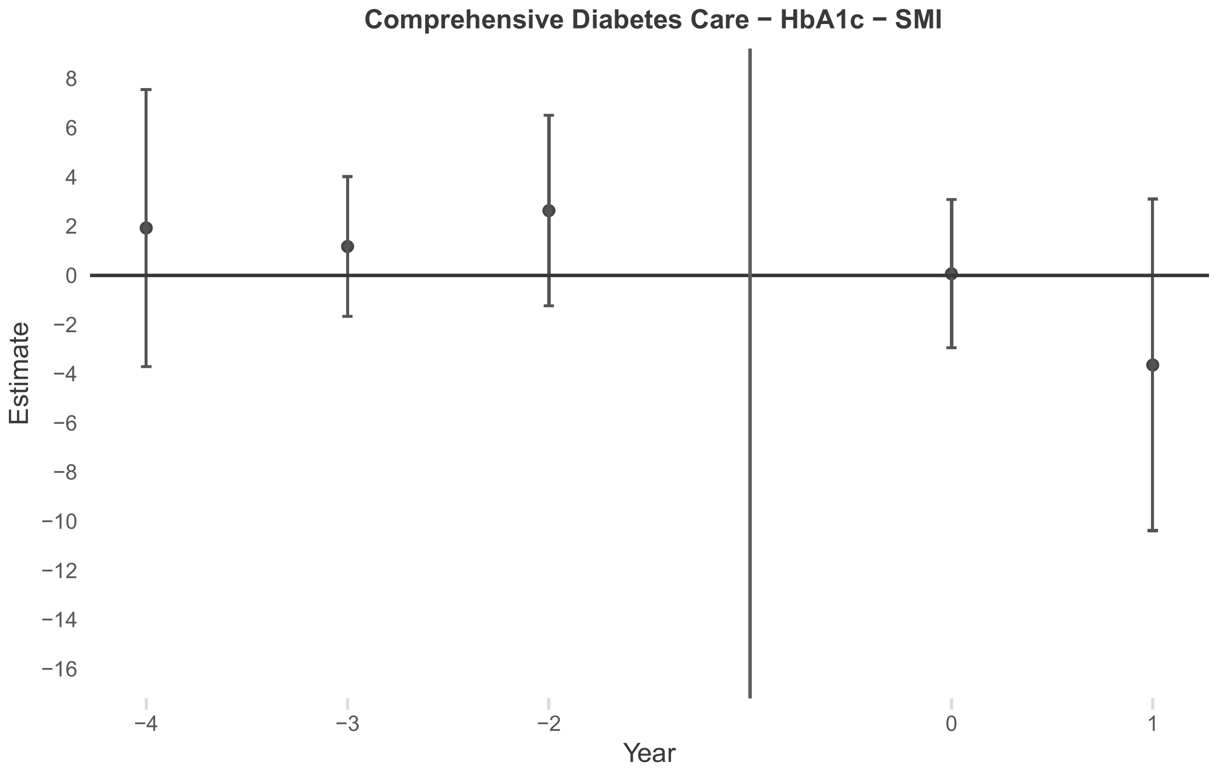

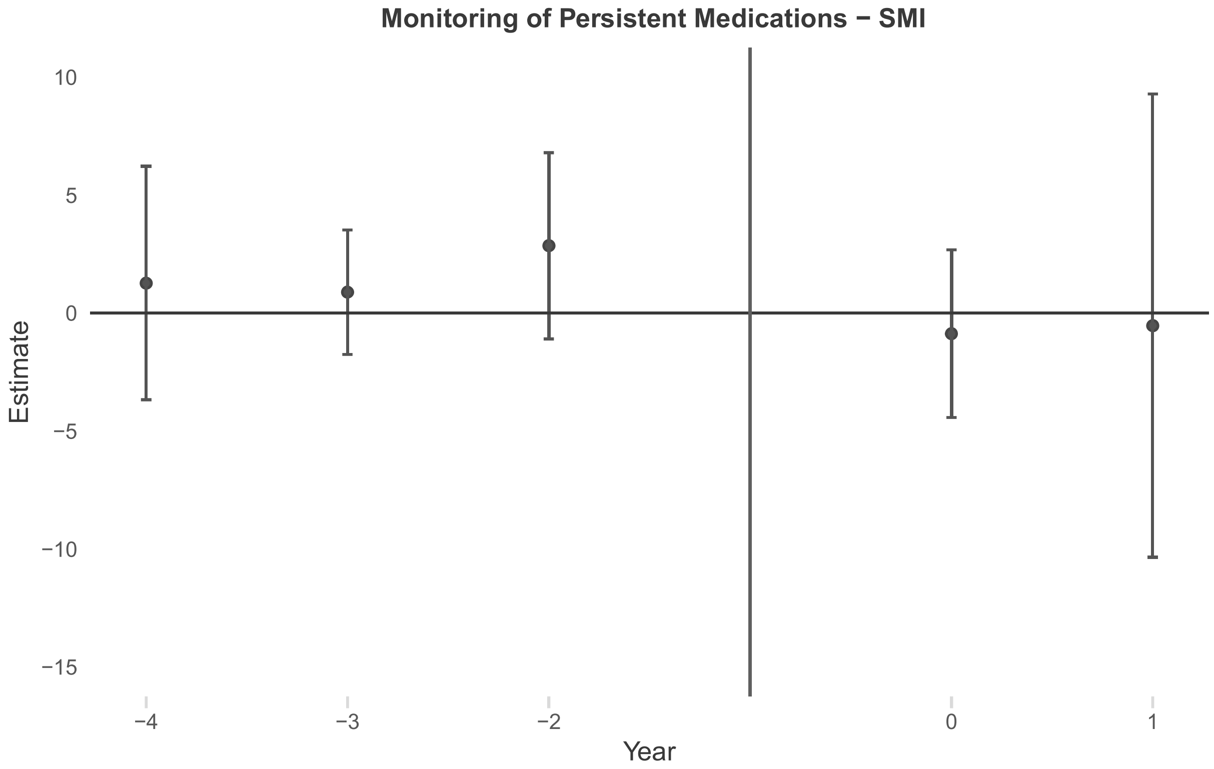

© 2023 McConnell KJ et al. JAMA Health Forum.

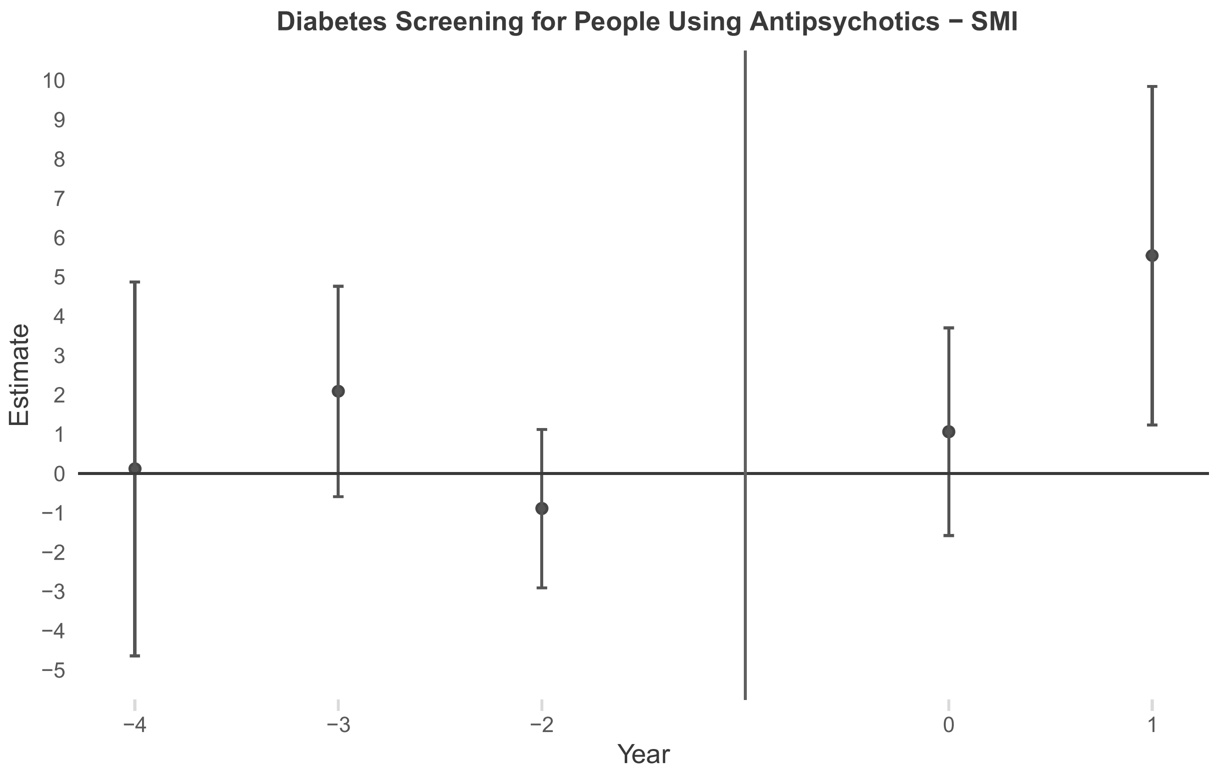

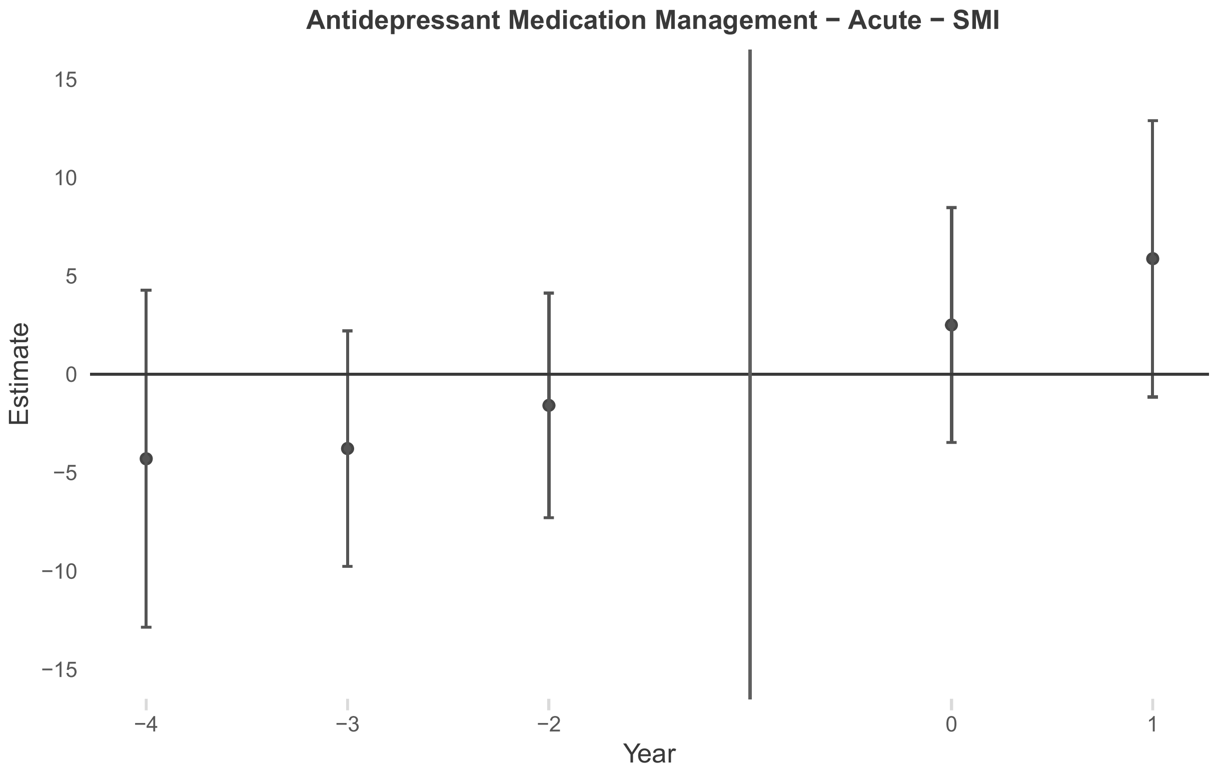

© 2023 McConnell KJ et al. JAMA Health Forum.

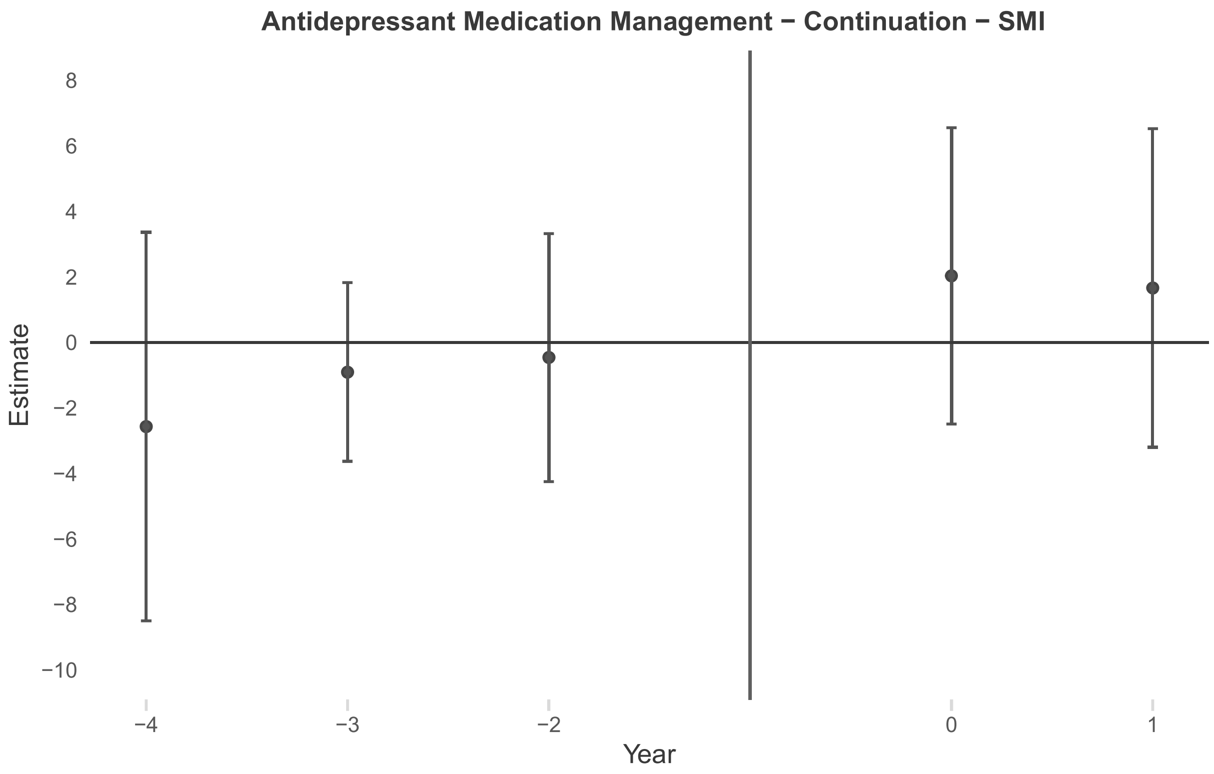

© 2023 McConnell KJ et al. JAMA Health Forum.

eMETHODS 3 Sensitivity Analyses

Group 1 included, no de-trending

Utilization Outcomes from Difference-in-Differences Analyses

Measure	SMI		Mild and moderate mental illness		No mental illness	
	Baseline	Estimate	Baseline	Estimate	Baseline	Estimate
Outpatient Mental Health Visits	811.1	-60.1 (-127.4, 7.2)	312.2	-6.3 (-31.1, 18.4)	0.7	-0.12 (-0.25, 0.02)
Primary Care Visits - Mental Health	56.7	0.4 (-8.2, 9.0)	46.9	1.3 (-0.5, 3.2)	1.8	-0.06 (-0.19, 0.08)
Primary Care Visits - Physical Health	305.3	-5.5 (-12.5, 1.4)	260.8	-1.0 (-6.2, 4.1)	151.2	-0.3 (-3.4, 2.8)
ED Visits for Mental Health Conditions	17.7	0.2 (-2.0, 2.4)	3.8	-0.0 (-0.3, 0.2)	0.2	0.01 (-0.09, 0.11)
Hospital-izations for Mental Health Conditions	16.0	0.1 (-3.3, 3.5)	1.0	0.3 (-0.1, 0.7)	NA	NA

* $P < 0.05$

Unit of observation is the person quarter. Measures are presented as rates per 1,000 member months

N = 1,200,899 for no mental illness, 290,095 for MMI, 72,066 for SMI

NOTE: The “No mental illness” population was defined as enrollees without at least one inpatient visit with a mental health diagnosis or at least other outpatient visits with a mental health diagnosis. Thus, it is possible for enrollees classified as having “No mental illness” to still have an outpatient mental health visit, a primary care mental health visit, or an ED visit for a mental health condition.

© 2023 McConnell KJ et al. JAMA Health Forum.

Group 1 included, no de-trending

Health, SDOH, and Quality Outcomes from Difference-in-Differences Analyses

		SMI		Mild and moderate mental illness	
		Baseline	Estimate	Baseline	Estimate
Health Out-comes	Read-mission after Mental Health Hospitalization	24.9	**-6.46* (-11.84, -1.08)**	10.9	1.3 (-2.3, 4.8)
	Self-Harm Incidents	4.4	0.45 (-0.07, 0.98)	1.0	-0.1 (-0.3, 0.1)
	Cardiac Events (IP or ED)	8.9	0.6 (-0.7, 2.0)	5.2	**-0.88* (-1.36, -0.40)**
	Hospital-izations for Diabetes	60.2	6.8 (-13.8, 27.4)	36.8	-12.4 (-28.6, 3.7)
SDOH-related	Employed	30.4	**-0.64* (-1.22, -0.06)**	41.5	**-0.73* (-1.29, -0.16)**
	Arrested	4.3	0.1 (-0.4, 0.5)	3.0	0.0 (-0.2, 0.2)
	Homeless	14.9	-0.2 (-0.6, 0.3)	9.0	-0.1 (-0.4, 0.1)

* P < 0.05

Unit of observation for Utilization, Health Outcomes, and SDOH Measures is the person quarter. Measures are presented as rates per 1,000 member months, with one exception: Hospitalizations for Diabetes are shown as rates per 100,000 member months. The unit of observation for Quality Measures is the person year.

© 2023 McConnell KJ et al. JAMA Health Forum.

Group 1 not included, no de-trending

Utilization Outcomes from Difference-in-Differences Analyses

Measure	SMI		Mild and moderate mental illness		No mental illness	
	Baseline	Estimate	Baseline	Estimate	Baseline	Estimate
Outpatient Mental Health Visits	805.6	-60.3 (-132.4, 11.8)	310.7	-18.1 (-47.9, 11.7)	0.7	-0.13 (-0.31, 0.05)
Primary Care Visits - Mental Health	57.4	-2.0 (-10.8, 6.9)	47.2	0.6 (-1.2, 2.5)	1.7	-0.08 (-0.25, 0.09)
Primary Care Visits - Physical Health	307.9	-3.8 (-16.4, 8.8)	262.0	-1.4 (-8.8, 6.1)	152.9	-1.7 (-5.9, 2.5)
ED Visits for Mental Health Conditions	17.7	0.3 (-2.2, 2.9)	3.8	-0.2 (-0.6, 0.2)	0.2	0.0 (-0.1, 0.1)
Hospital-izations for Mental Health Conditions	15.8	2.4 (-3.5, 8.2)	0.9	0.41 (-0.08, 0.90)	NA	NA

* $P < 0.05$

Unit of observation is the person quarter. Measures are presented as rates per 1,000 member months.

N = 1,115,332 for no mental illness, 271,350 for MMI, 67,503 for SMI

NOTE: The “No mental illness” population was defined as enrollees without at least one inpatient visit with a mental health diagnosis or at least other outpatient visits with a mental health diagnosis. Thus, it is possible for enrollees classified as having “No mental illness” to still have an outpatient mental health visit, a primary care mental health visit, or an ED visit for a mental health condition.

© 2023 McConnell KJ et al. JAMA Health Forum.

Group 1 not included, no de-trending

Health, SDOH, and Quality Outcomes from Difference-in-Differences Analyses

		SMI		Mild and moderate mental illness	
		Baseline	Estimate	Baseline	Estimate
Health Out-comes	Read-mission after Mental Health Hospitalization	23.8	-2.5 (-9.7, 4.7)	9.3	3.2 (-2.0, 8.4)
	Self-Harm Incidents	4.3	0.2 (-0.5, 1.0)	1.0	-0.1 (-0.3, 0.2)
	Cardiac Events (IP or ED)	9.1	0.2 (-1.6, 2.0)	5.1	**-0.79* (-1.40, -0.18)**
	Hospital-izations for Diabetes	63.7	13.0 (-16.9, 42.9)	38.1	-15.9 (-35.7, 3.9)
SDOH-related	Employed	30.9	**-0.87* (-1.56, -0.18)**	42.1	**-0.70* (-1.39, 0.00)**
	Arrested	4.3	0.3 (-0.1, 0.8)	3.1	0.1 (-0.1, 0.4)
	Homeless	15.2	-0.1 (-0.6, 0.4)	9.1	-0.1 (-0.4, 0.2)

* P < 0.05

Unit of observation for Utilization, Health Outcomes, and SDOH Measures is the person quarter. Measures are presented as rates per 1,000 member months, with one exception: Hospitalizations for Diabetes are shown as rates per 100,000 member months. The unit of observation for Quality Measures is the person year.

© 2023 McConnell KJ et al. JAMA Health Forum.

Group 1 included, de-trended

Utilization Outcomes from Difference-in-Differences Analyses

Measure	SMI		Mild and moderate mental illness		No mental illness	
	Baseline	Estimate	Baseline	Estimate	Baseline	Estimate
Outpatient Mental Health Visits	811.1	-21.2 (-88.5, 46.1)	312.2	1.8 (-23.0, 26.5)	0.7	-0.12 (-0.25, 0.01)
Primary Care Visits - Mental Health	56.7	-1.2 (-9.8, 7.4)	46.9	1.0 (-0.8, 2.9)	1.8	-0.10 (-0.23, 0.03)
Primary Care Visits - Physical Health	305.3	-1.7 (-8.6, 5.2)	260.8	2.4 (-2.7, 7.5)	151.2	0.0 (-3.1, 3.1)
ED Visits for Mental Health Conditions	17.7	-0.1 (-2.2, 2.1)	3.8	0.0 (-0.2, 0.3)	0.2	-0.01 (-0.11, 0.09)
Hospital-izations for Mental Health Conditions	16.0	-0.8 (-4.2, 2.6)	1.0	0.37 (-0.05, 0.78)	NA	NA

* P < 0.05

Unit of observation is the person quarter. Measures are presented as rates per 1,000 member months.

N = 1,200,899 for no mental illness, 290,095 for MMI, 72,066 for SMI

NOTE: The “No mental illness” population was defined as enrollees without at least one inpatient visit with a mental health diagnosis or at least two other visits with a mental health diagnosis. Thus, it is possible for enrollees classified as having “No mental illness” to still have an outpatient mental health visit, a primary care mental health visit, or an ED visit for a mental health condition.

© 2023 McConnell KJ et al. JAMA Health Forum.

Group 1 included, de-trended

Health, SDOH, and Quality Outcomes from Difference-in-Differences Analyses

		SMI		Mild and moderate mental illness	
		Baseline	Estimate	Baseline	Estimate
Health Out-comes	Read-mission after Mental Health Hospitalization	24.9	**-7.50* (-12.88, -2.12)**	10.9	2.5 (-1.1, 6.1)
	Self-Harm Incidents	4.4	0.2 (-0.3, 0.8)	1.0	-0.10 (-0.30, 0.09)
	Cardiac Events (IP or ED)	8.9	0.6 (-0.7, 2.0)	5.2	**-1.01* (-1.49, -0.54)**
	Hospital-izations for Diabetes	60.2	7.9 (-12.7, 28.5)	36.8	-15.3 (-31.5, 0.8)
SDOH-related	Employed	30.4	**-0.61* (-1.19, -0.03)**	41.5	**-0.57* (-1.13, 0.00)**
	Arrested	4.3	0.3 (-0.2, 0.7)	3.0	0.14 (-0.06, 0.33)
	Homeless	14.9	0.2 (-0.2, 0.7)	9.0	-0.0 (-0.3, 0.2)

* P < 0.05

Unit of observation for Utilization, Health Outcomes, and SDOH Measures is the person quarter. Measures are presented as rates per 1,000 member months, with one exception: Hospitalizations for Diabetes are shown as rates per 100,000 member months. The unit of observation for Quality Measures is the person year.

© 2023 McConnell KJ et al. JAMA Health Forum.
