# Supplementary material for: Access, Utilization, and Quality of Behavioral Health Integration in Medicaid Managed Care
Source: JAMA Health Forum. 2023 Dec 28;4(12):e234593. doi: 10.1001/jamahealthforum.2023.4593 (PMC10755612; doi:10.1001/jamahealthforum.2023.4593)
Supplement: Supplement 2. — Data sharing statement [file jamahealthforum-e234593-s002.pdf]

Data Sharing Statement

Data
Data available: No

Additional Information
Explanation for why data not available: Data are obtained from the State of Washington with Data Use Agreement and we are not permitted to make data available independently.
